# Injury Profile in Youth Female Athletes: A Systematic Review and Meta-Analysis

**DOI:** 10.1007/s40279-023-01988-w

**Published:** 2024-01-24

**Authors:** Jake Beech, Ben Jones, Thomas Hughes, Stacey Emmonds

**Affiliations:** 1 Carnegie School of Sport, Leeds Beckett University, Leeds, UK; 2 The Football Association, Burton-Upon-Trent, UK; 3 England Performance Unit, The Rugby Football League, Leeds, UK; 4 Leeds Rhinos Rugby League Club, Leeds, UK; 5 Division of Exercise Science and Sports Medicine, Department of Human Biology, Faculty of Health Sciences, the University of Cape Town and the Sports Science Institute of South Africa, Cape Town, South Africa; 6 School of Science and Technology, University of New England, Armidale, NSW Australia

## Abstract

**Background:**

An increasing number of epidemiological studies assessing the incidence, prevalence and severity of injury in youth female sport are available. However, no study has sought to synthesise the current evidence base across all youth female sport. As such, a systematic review and meta-analysis of injury in this cohort is necessary to understand the diversity of injury and its associated burden between sports in addition to identifying the density of research available.

**Objective:**

To conduct a systematic review and meta-analysis of epidemiological data of injuries in youth female athletes with particular attention to injury incidence, mean days lost and injury burden.

**Methods:**

Searches were performed in PubMed, EBSCO (SPORTDiscus with Full Text MEDLINE, APA PsycINFO, CINAHL, Academic Search Complete) and Cochrane databases. Studies were considered if they reported time-loss injury incidence or prevalence in youth female (≤ 19 years old) athletes. Study quality and risk of bias were assessed using STROBE-SIIS extension, Newcastle-Ottawa Scale, and funnel plots, respectively. Injury incidence and burden rate data were modelled using a mixed-effect Poisson regression model. Days lost data were modelled using a generalised linear mixed model.

**Results:**

Thirty-two studies were included. The overall incidence rate, mean days lost per injury, and burden rate were 4.4 injuries per 1000 h (95% CI 3.3–5.9), 10 days (95% CI 6–15), and 46 days per 1000 h (95% CI 23–92), respectively. Forty percent of athletes sustained at least one time-loss injury. Competitive level was a significant moderator of match and training injury incidence, with elite youth athletes presenting greater pooled injury incidence estimates than non-elite athletes (*p* = 0.0315 and *p* = 0.0047, respectively). The influence of moderators on days lost and injury burden could not be determined due to an insufficient number of studies for analysis.

**Conclusion:**

Despite a broad inclusion criterion, there is limited injury surveillance research available across youth female sport. Outside of soccer, little research density is evidenced with single studies available in popular team sports such as Australian football and rugby union. Insufficient study numbers reporting mean days lost and injury burden data were available for analysis, and pooled days lost data could only be estimated for soccer. This highlights a need for future research to report days lost data alongside injury number and exposure so burden can be calculated and the full risk of injury to youth female athletes can be identified.

**Supplementary Information:**

The online version contains supplementary material available at 10.1007/s40279-023-01988-w.

## Key Points

**Table:** 

Competitive level was a significant moderator of match and training injury incidence with elite youth female athletes presenting greater injury incidence rates than non-elite athletes.
An insufficient number of studies reporting mean days lost and injury burden were available for analysis. Future surveillance research in youth female sport should make an effort to report these variables alongside injury incidence.

## Introduction

Youth female sport has experienced an exponential growth in recent years. Sport participation provides physiological (e.g., increased aerobic fitness, strength, [1]) and psychological (e.g., development of self-esteem, peer socialization, team play [2]) benefits in children and adolescents. However, sport participation also carries an inherent risk of injury, with a reported 30–40% of injuries in children and adolescents occurring during sport participation [1]. An increasing number of epidemiology studies assessing injury incidence, prevalence, risk factors and injury prevention measures are available in youth female sport. Overall injury incidence rates (number of injuries per 1000 h of exposure) of youth female soccer athletes range from 4.6 to 9.9 injuries per 1000 h, with a range of 17–45 injuries per 1000 h present in youth female rugby union [3]. Fewer injury surveillance studies are present in popular team sports such as basketball, volleyball, handball, cricket, and field and ice hockey [3], and in individual sports such as track and field and tennis. To the authors’ knowledge, no study has sought to combine and meta-analyse epidemiological data in youth female athletes aged ≤ 19 years across team and individual sports. Whilst a number of systematic reviews and meta-analyses are available in youth female athletes [3–5], these have been conducted in single sport settings or sport types (i.e., team sport athletes only) meaning the diversity of injury and associated burden across different sports (team sports vs. racket sports, etc.) is poorly understood.

Developing a full understanding of injury in a given sport is essential to inform management strategies that can reduce the impact of injury in youth athletes [6, 7], improving their health status in addition to facilitating athletic development and performance [8]. Therefore, it is important that descriptive epidemiological studies capture all injuries and report on injury incidence, severity (the number of days the athlete is unavailable for un-modified training and competition) [9, 10], and associated burden (the number of days lost per 1000 h of exposure) [11, 12]. Indeed, severe injuries (e.g., complete anterior cruciate ligament ruptures) [13, 14] in addition to frequent and less severe traumatic (acute) injuries to soft tissues (e.g., hamstring muscle strains) and overuse injuries (e.g., stress fractures) present significant burden to youth female athletes [2, 15, 16]. Furthermore, whilst injury incidence and severity are essential metrics and often reported in injury research, when reported in isolation of each other they can present an erroneous picture of injury risk [11]. The cross-product of both, injury burden, allows for a more thorough assessment, and provides direction to management strategies for injuries with the greatest consequence to athletes [11]. Conducting a systematic review and meta-analyses of injury data in youth female athletes aged ≤ 19 years participating in multiple sports with reference to injury incidence, severity and associated burden would allow for a detailed understanding of injury in youth female sport and direct comparisons between sports. Furthermore, this review would also demonstrate any potential gaps in the literature from contextual (i.e., the density of research available in given sports or not) and methodological (i.e., what injury variables are reported in the available literature) perspectives, providing direction for future surveillance studies in youth female sport.

## Objectives

The purpose of this review was to meta-analyse the epidemiological data of time-loss injuries in youth female athletes with particular reference to injury incidence rates, injury severity and injury burden rates. Additionally, the proportion of injuries as a function of severity, body region, location, type, onset and mechanism are summarised. The effect of sport, in addition to other circumstances that influence injury outcomes such as age, competitive level, injury recording method and competition type, was also explored.

## Methods

Preferred Reporting Items for Systematic Reviews and Meta-Analysis (PRISMA) guidelines [17] were followed. Details of the protocol for this systematic review were registered with PROSPERO (registration number: CRD42021290401.) and can be accessed at www.crd.york.ac.uk/prospero/display_record.php?RecordID=290401.

### Literature Search

PubMed, EBSCO (SPORTDiscus with Full Text MEDLINE, APA PsycINFO, CINAHL, Academic Search Complete) and Cochrane databases were searched (by JB) for articles published before 15 December 2022 using the search terms and search strategy presented in the Online Supplementary Material (OSM) Table S1. Additionally, the reference lists of retrieved studies and relevant conference proceedings, presentations and injury surveillance reports were manually searched to identify additional articles. The inclusion criteria for retrieved studies were: (1) prospective cohort studies or randomised controlled trials where the control group can be distinguished; (2) study population comprising female athletes under the age of 19 years; (3) studies that include male youth athletes where the female group is distinguishable and data can be readily extracted; (4) full-text version available in English; (5) injury defined as time-loss (i.e., an injury resulting in an athlete being unable to take a full part in future training and competition/match-play); and (6) reports injury incidence rates per 1000 h of exposure and/or the prevalence of injury amongst the surveyed population plus one or more of the following injury variables: (1) days lost due to injury; (2) severity time-bins; (3) injury location or type; (4) mechanism of injury; (5) exposure hours or provide sufficient data in figures for these variables to be calculated. Details of the inclusion and exclusion criteria for retrieved studies are presented in Table 1. Duplicates were identified and removed, and the titles and abstracts of the remaining studies were assessed by JB and TH, with non-relevant studies being removed. Full-text versions of the outstanding articles were then retrieved and evaluated against the inclusion criteria by two independent reviewers (JB and TH). All conflicts of inclusion were resolved between the two reviewers and SE.

**Table 1:** Study inclusion and exclusion criteria

Criterion	Inclusion	Exclusion
	Definition	Definition
Population	Injury surveillance conducted in female athletes ≤ 19 years Studies that include male youth athletes where the female group is distinguishable, and data can be readily extracted	Injury surveillance conducted in men and/or youth males only or in females ≥ 19 years Injury surveillance conducted in samples less than five athletes
Outcome	Injury defined as time-loss Injury incidence per 1000 h of exposure and/or injury prevalence reported or sufficient data present in tables and figures to be calculated	Non-time loss injury definition Injury incidence and/or prevalence amongst surveyed athletes not reported or insufficient data in tables and figures to be calculated Injury surveillance concerning single injury type, body region/location and onset
Study design	Prospective cohort study or randomised controlled trials whereby control group can be distinguished Full text original primary article in English published in a peer-reviewed journal before data extraction	Expert opinions, review(s), case report, current concept, cross-sectional design or retrospective design Non-English language studies

### Data Extraction

In addition to general study information (author, year, title, study design, injury definition used), the following data were extracted (by JB) for studies meeting the inclusion criteria: (1) study population characteristics (sample size, age, biological maturity status, morphological characteristics, sport, performance level); (2) type of competition (season and tournament); (3) exposure hours (overall, competition and training); (4) injury recording method (self-reported, non-medical or medical); and (5) injury characteristics (number of injury events; number of injured athletes and prevalence of injury amongst surveyed athletes; total, mean and/or median days lost per injury). The number/proportion of injuries as a function of severity time-bin, location, type, onset and mechanism was also extracted. Where necessary, the authors of included studies were contacted to provide clarifications and/or access to raw data. Where injury count, exposure data or incidence rate were not provided, the missing component of the three variables was calculated using the data available (e.g., missing exposure data calculated as: (injury count/injury incidence) × 1000). The same process was applied for unreported days lost data if applicable (e.g., missing mean severity calculated as: injury count/total days lost). Note that small rounding errors may occur as a result of the calculation; however, these errors have a negligible impact on reporting outcomes [18]. Where applicable, study operational definitions of injury location, type, mechanism and onset were aligned with those of injury consensus statements [10]. To increase comparability between studies, injury severity time-bins diverged from those of injury consensus statements [10]. Minor injuries were defined as injuries resulting in 1–7 days lost, with moderate and severe injuries requiring 8–28 days and greater than 28 days lost, respectively.

Injury recording methods were classified into one of three labels: self-reported, non-medical and medical. Self-reported was defined as methods whereby the player self-reports an injury via a questionnaire or SMS messaging platform. Non-medical included methods whereby a non-qualified designated individual(s)/third party collected injuries as they happened and were present at exposure events. Medical included qualified medical practitioners collecting injuries as they happened and were present at exposure events. If a range of different methods were used to record injury these were deemed as mixed. No distinctions were made for methods of diagnosis as the influence of diagnosis methods on injury diagnoses is out of the scope of this review. Female youth athletes were classified into one of three labels: child, adolescent, or child and adolescent. Children were defined as players aged between 6 and 12 years and adolescents were defined as players aged between 13 and 19 years. For studies that covered both child and adolescent age ranges, the child and adolescent group was applied. Additionally, youth female athletes were classified into one of two competitive level groups: sub-elite or elite. Elite youth athletes were defined as athletes between the age of 8 and 19 years whose performance status was described in studies as “elite”, “high-level”, “national level”, “international level” or being part of an “academy” or a “performance pathway” [4]. Players not described in studies as “elite”, “high-level”, “national level”, “international level” or as being part of an “academy” or a “performance pathway” were considered to be non-elite [4].

### Assessment of Reporting Quality and Risk of Bias

The reporting quality of studies included for meta-analysis was assessed using the ‘Strengthening the Reporting of Observational Studies in Epidemiology’ (STROBE) Sports Injury and Illness Surveillance (SIIS) statement [10]. Although not intended as a direct assessment of study quality, this 23-item checklist provides guidance on the reporting of observational studies on injury and illness in sporting contexts and has been used by previous meta-analyses investigating the epidemiology of injury in rugby union [18]. An adapted version of the Newcastle-Ottawa Scale (NOS) for cohort studies was used to assess the risk of bias of external validity quality. This tool was chosen as it has been highlighted as the most appropriate for cohort studies [19]. Similar adaptations of the NOS scale have been employed by previous meta-analyses investigating the epidemiology of injuries in other cohorts of athletes including senior female soccer [20] and senior male soccer players [21]. Additionally, the risk of small study bias was examined visually through funnel plots.

### Statistical Analysis and Interpretation of Results

All statistical analysis was performed in R (version 4.1.3, R Foundation for Statistical Computing, Vienna, Austria) using the *metafor* package [22]. Injury outcomes were (1) all injuries; (2) match injuries; (3) training injuries; (4) total days lost due to all injuries; (5) total days lost due to match injuries; and (6) total days lost due to training injuries. Incidence and burden rates data were modelled using a mixed-effects Poisson regression model. For injury incidence rate and injury burden rate models, the response variable was the number of injuries and total numbers of days lost due to injury, offset by the log of the number of exposure hours, respectively. Days lost were modelled using a general linear mixed model [22]. Injury location, type and severity time-bin, onset and mechanism were summarised as a proportion of all injuries in a given study, and then analysed using random effects models with raw proportions. Between-study heterogeneity was evaluated using the *I*^2^ statistic and categorised as low, moderate and high for values of 25%, 50% and 75%, respectively [23]. High levels of heterogeneity were observed in all injury outcomes, and thus random-effects term was included in all models to account for the correlation arising from using multiple rows of data in the same study. A pooled estimate was calculated for each outcome with two or more studies and summarised in a forest plot. Subgroup analyses were performed for each outcome for which ten or more studies were available [24]. To account for the potential influence of sport on pooled estimates, it was included as a fixed effect in all models. False discovery rate post hoc pairwise comparisons for differences between sport estimates were conducted using the *glht* function (*multcomp* package version 1.4-18) if sport was a significant moderator in the model. Additionally, the influence of potentially confounding methodological moderators were analysed. These were methodological characteristics that could potentially influence the completeness and validity of data in addition to leading to an increased risk of bias, including (1) type of competition (season vs. tournament), (2) competitive level (elite vs. non-elite), (3) age group (child vs. adolescent), (4) injury data collection method (medical vs. non-medical vs. self-reported), and (5) study quality (≥ 15 STROBE-SIIS rating vs. < 15 STROBE-SIIS rating, cut-off representing the median STROBE-SIIS rating). All estimates are reported with corresponding 95% confidence intervals (CIs). Statistical significance was set at an alpha level of 0.05.

## Results

### Search Results

**Fig. 1:**
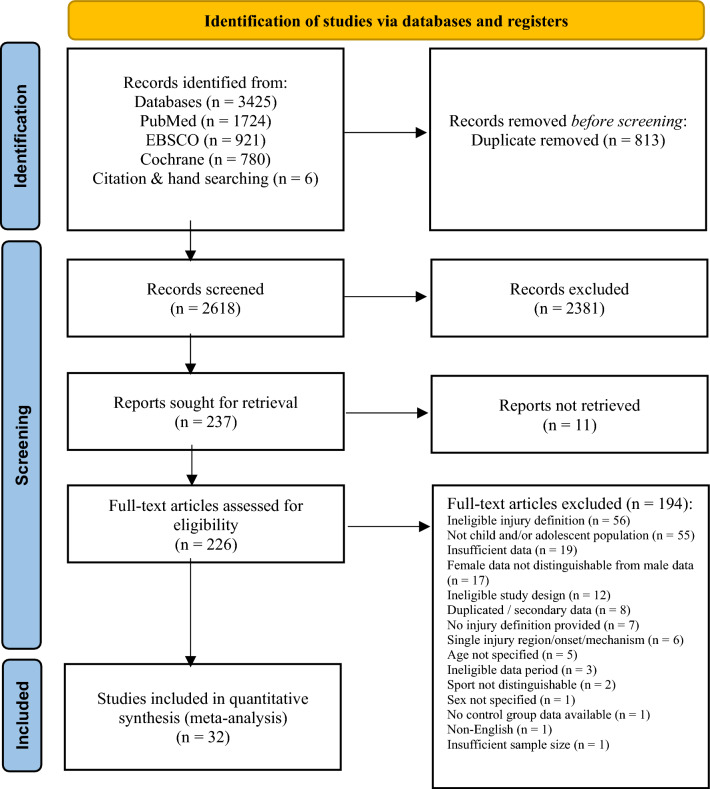
PRISMA flow diagram for study selection process

The search of electronic databases returned 3431 references. Of those, 813 were removed as duplicates (24%). A further 2381 studies were excluded after reading the title and abstract. After full-text screening of 226 studies, 194 studies were excluded because they (1) employed an ineligible injury definition (non-time-loss definition or substantial injuries that caused > 1 week absence); (2) were not conducted in a child or adolescent population (> 19 years old); (3) did not report required injury data; (4) did not distinguish female injury data from male data; (5) collected data retrospectively or employed a cross-sectional design; (6) reported secondary injury data; (7) did not provide a definition of injury; (8) only reported single injury onset, region or mechanisms; (9) did not specify the age of the surveyed athletes; (10) did not collect injury data over the entire course of a season or tournament; (11) did not distinguish injury data from different sports; (12) did not specify the sex of the athletes surveyed; (13) did not report injury data for the control group of a randomised controlled trial; (14) were non-English studies; or (15) collected data in samples of less than five athletes. Finally, data from the remaining 32 studies were included in the qualitative and quantitative analysis (Fig. 1). Nineteen of the 32 authors were contacted for additional data. Eight of the authors contacted gave additional details, when requested [25–32].

### Characteristics of Included Studies and Methodological Quality and Bias

Included studies collected injury and exposure data between 1995 and 2020 and publication dates ranged from 1999 to 2022. Selected studies comprised child and adolescent females participating in soccer (elite [30, 33, 34], high-level [32], amateur [26, 35–40] soccer leagues and world [28, 41], European [42] and national [43] tournaments), handball [25, 44, 45], tennis [31, 46, 47], track and field [48–50] rugby union [26], Australian Rules football [51], floorball [52], volleyball [29, 53], cricket [27], ice hockey [54], alpine skiing [55] and basketball [56] (Table 2). Of these studies, five were from England [26, 27, 30, 33, 48], four from Sweden [46, 52, 55, 57], three each from the USA [29, 38, 53] and Canada [36, 54, 56], two each from Australia [47, 51], Norway [39, 40] and Denmark [35, 44], and one each from Chile [49], Finland [32], France [34], Germany [25], Ireland [37] and Kenya [43]. Twenty-seven studies presented in-season data [25–27, 29–40, 44, 46–56], with the remaining five studies presenting tournament data (national tournaments, European Championships and World Championships) [28, 41–43, 45]. The majority of studies investigated adolescent athletes (*n* = 23) [25–30, 34–37, 39–42, 44–48, 50, 52, 53, 55], and nine studies covered children and adolescent age ranges [31–33, 38, 43, 49, 51, 54, 56]. Twenty-nine studies [25–27, 29–36, 38–40, 42–56] collected data from a total of 15,908 youth female athletes. Three studies [28, 37, 41] presented no information on sample size. Of the 29 studies that reported injury incidence [25, 26, 28, 30–52, 54–56], ten [26, 28, 30, 32–34, 36, 47, 48, 51] reported information on days lost due to injury (mean days lost and injury burden). Twelve studies reported injury prevalence amongst surveyed athletes [26, 27, 29, 31, 34, 38, 42, 45, 46, 50, 53, 55]. Details regarding sample characteristics, injury data, and exposure data for each study are presented in Table 2. The majority of studies collected injury data via medical practitioners (*n* = 16, [25–30, 33, 34, 37, 41–43, 47, 49, 53, 54]), followed by self-reported (*n* = 9, [31, 32, 35, 44, 46, 48, 50, 52, 55]) and non-medical personnel (*n* = 4, [36, 39, 40, 45]) methods. Three studies employed a mixed-methods approach [38, 51, 56]. Of these, two utilised both medical and non-medical practitioners to collect injury [38, 51], with Owoeye et al. [56] employing athlete self-reporting and medical and non-medical personnel methods. Fourteen studies were conducted in elite youth sport settings [27, 28, 30–34, 41, 42, 44, 47, 48, 50, 53] with 17 studies investigating injury epidemiology in non-elite cohorts [25, 26, 29, 35–40, 43, 45, 46, 49, 52, 54–56]. Farley et al. [51] assessed injury in both elite and non-elite youth female Australian Rules football players. The mean ± SD reporting quality, as assessed by the 23-item STROBE-SIIS checklist, was 15 ± 2 with a range of 11–19. The mean ± SD risk of bias, as assessed by the eight-item NOS criteria, was 7 ± 1 with a range of 5–8. Each individual rating for the STROBE-SIIS and NOS is given in OSM Tables S2 and S3, respectively. Visual inspection of funnel plots indicated no small-study publication bias (Fig. S1, OSM).

**Table 2:** Study characteristics, injury data, STROBE-SIIS rating and NOS rating of included studies

References	Setting	Sport	Level	Surveillance period	Athletes,* n*	Age range, y	Activity	Injury count	Injured athletes no. (% of athletes surveyed)	Exposure time (h)	Incidence (no./1000 h)	Mean days missed	Total days missed	Burden (days/1000 h)	STROBE-SIIS rating [/23]	NOS rating [/8]
*Prospective season*																
Achenbach et al. [25]	Germany	Handball	Organised	2015/2016	76	U16	Overall	17	–	12,322	1.4	–	–	–	13	6
							Match	7	–	1156	6.1	–	–	–		
							Training	10	–	11,166	0.9	–	–	–		
Akerlund et al. [52]^a^	Sweden	Floorball	Organised	2017/2018	35	U14	Overall	9	–	1875	4.8	–	–	–	17	7
Barden et al. [26]	England	Soccer	Organised	2016–2019	83	16–19	Match	20	15 (75)	940	21.3	25	477	507	13	5
		Rugby Union	Organised	2016–2019	54	16–19	Match	30	17 (57)	562	53.4	40	1353	2407		
Beech et al. [33]	England	Soccer	Elite	2019/2020	62	U10	Overall	3	–	7347	0.4	39	117	16	16	6
							Match	1	–	706	1.4	96	96	136		
							Training	2	–	6641	0.3	11	21	3		
					104	U12	Overall	15	–	12,813	1.2	12	177	14		
							Match	7	–	1563	4.5	21	144	92		
							Training	6	–	11,250	0.5	4	33	3		
					104	U14	Overall	40	–	16,076	2.5	13	516	32		
							Match	21	–	1919	10.9	12	249	130		
							Training	19	–	14,158	1.3	14	267	19		
					105	U16	Overall	53	–	16,598	3.2	22	1136	68		
							Match	30	–	2185	13.7	23	678	310		
							Training	23	–	14,413	1.6	20	458	32		
Clausen et al. [35]	Denmark	Soccer	Organised	2012	498	15–18	Overall	269	–	27,746	9.7	–	–	–	14	7
							Match	123	–	6285	19.6	–	–	–		
							Training	49	–	21,461	2.3	–	–	–		
Decloe et al. [54]	Canada	Ice Hockey	Organised	2008/2009	324	9–17	Overall	44	–	27,744	1.6	–	–	–	16	7
Emery et al. [36]		Soccer	Organised	13 weeks	164	U14–U18	Overall	30	–	6942	4.3	12	347	50	15	7
							Match	19	–	3509	5.4	9	259	74		
							Training	4	–	3433	1.2	3	88	26		
Farley et al. [51]	Australia	Australian Rules football	School or community	2017/2018–2018/2019	33	10–13	Overall	12	–	2109	5.7	26	314	149	19	6
							Match	9	–	580	15.5	33	298	514		
							Training	3	–	1529	2	5	16	10		
			School or community	2017/2018–2018/2019	29	14–17	Overall	4	–	2193	1.8	15	60	27		
							Match	2	–	462	4.3	12	23	50		
							Training	2	–	1731	1.2	19	37	21		
			High-level	2017/2018–2018/2019	33	U18	Overall	49	–	3724	13.2	17	848	228		
							Match	28	–	582	48.1	18	492	845		
							Training	21	–	3142	6.7	21	356	113		
Gescheit et al. [47]	Australia	Tennis	Elite	2012–2016	43	13–18	Overall	258	–	92,143	2.8	1	284	3	17	6
Goggins et al. [27]	England	Cricket	International	2015–2019	52	14–19	Overall	35	23 (66)	–	–	6	208	–	16	6
Hjelm et al. [46]	Sweden	Tennis	Organised	2 years	20	15.4	Overall	27	14 (52)	45,000	0.6	–	–	–	14	7
Horan et al. [37]	Ireland	Soccer	Organised	2018–2019	–	U17	Match	2	–	318	6.3	–	–	–	15	7
Jacobsson et al. [50]	Sweden	Track and Field	Elite	52 weeks	71	17	Overall	85	21 (30)	–	–	–	–	–	17	7
							Competition	26	–	–	–	–	–	–		
							Training	59		30,743	1.9	–	–	–		
Le Gall et al. [34]	France	Soccer	Elite	1998–2008	119	15–19	Overall	619	110 (18)	97,325	6.4	18	1142	12	14	5
							Match	219	–	9795	22.4	–	–	–		
							Training	400	–	87,530	4.6	–	-	–		
Mann et al. [48]	England	Distance Running	Club and International	24 weeks	73	13018	Overall	63	–	4504	14	8	530	118	17	8
McGuine et al. [29]	USA	Volleyball	High School	2018	2072	14–18	Overall	393	354 (90)	–	–	–	–	–	14	6
							Match	163	160 (98)	–	–	–	–	–		
							Training	230	211 (92)	–	–	–	–	–		
Mendez-Rebolledo et al. [49]^a^	Chile	Track and Field	High School	March–June 2019	11	11–18	Overall	21	–	1174	17.9	–	–	–	12	6
Moller et al. [44]	Denmark	Handball	Elite	31 weeks	89	U16	Overall	117	–	17,320	6.8	–	–	–	14	8
							Match	15	–	1385	10.8	–	–	–		
							Training	47	–	15,935	2.9	–	–	–		
					53	U18	Overall	50	–	10,705	4.7	–	–	–		
							Match	11	–	845	13	–	–	–		
							Training	21	–	9860	2.1	–	–	–		
Owoeye et al. [56]	Canada	Basketball	High School and organised	8–14 weeks	200	11–18	Overall	46	14 (52)	45,000	3.7	–	–	–	17	6
Pluim et al. [31]	Netherlands	Tennis	Elite	32 weeks	28	9–14	Overall	32	21 (75)	7095	4.5	–	–	–	15	6
Schiff et al. [38]	USA	Soccer	Organised	1 season	80	12–14	Overall	27	22 (81)	5745	4.7	–	–	–	13	6
Sokka et al. [32]^a^	Finland	Soccer	Talented	2015	163	9–14	Overall	74	–	12,354	6	7	488	40	16	7
							Match	41	–	1700	24.1	9	353	207		
							Training	33	–	10,654	3.1	4	139	13		
Soligard et al. [40]^a^	Norway	Soccer	Organised	2007	837	13–17	Overall	215	–	45,428	4.7	–	–	–	18	7
							Match	138	–	14,342	9.6	–	–	–		
							Training	74	–	31,086	2.4	–	–	–		
Sprouse et al. [30]	England	Soccer	International	2012–2020	5852	15–19	Overall	326	–	51,150	6.4	18	6021	118	15	6
							Match	133	–	4989	26.7	21	2811	563		
							Training	193	–	46161	4.2	17	3210	70		
Steffen et al. [39]^a^	Norway	Soccer	Organised	2005	947	U17	Overall	241	–	65,725	3.7	–	–	–	16	8
							Match	151	–	19,856	7.6	–	–	–		
							Training	59	–	45,869	1.3	–	–	–		
Watson et al. [53]	USA	Volleyball	Interscholastic	1 season	2073	14–18	Overall	203	187 (92)	–	–	–	–	–	14	6
Westin et al. [55]	Sweden	Alpine Skiing	Competitive	5 years	216	16.7	Overall	162	102 (63)	91,525	1.8	–	–	–	14	7
*Prospective tournament*																
Hagglund and Walden [42]	UEFA European Championships	Soccer	International	2006	144	U19	Overall	23	21 (91)	1707	13.5	15	345	202	15	7
							Match	14	–	497	28.2	–	–	–		
							Training	9	–	1210	7.4	–	–	–		
				2007	144	U19	Overall	12	11 (92)	1407	8.5	8	96	68		
							Match	11	–	501	22	–	–	–		
							Training	1	–	906	1.1	–	–	–		
				2008	145	U19	Overall	8	7 (88)	1635	4.9	5	40	24		
							Match	6	–	514	11.7	–	–	–		
							Training	2	–	1121	1.8	–	–	–		
Junge and Dvorak [41]	U17 Women's World Cup	Soccer	International	2008, 2010 and 2012	–	U17	Match	68	–	3168	21.5	3	203	64	13	6
Junge and Dvorak [28]	U19 Women's World Championship	Soccer	International	World championship 2002	–	U19	Match	48	–	858	55.9	–	–	–	15	6
				World championship 2004	–	U19	Match	17	–	858	19.8	–	–	–		
Lislevand et al. [43]	Kenya	Soccer	Amateur	2–day tournament	433	U13	Match	5	–	431	11.6	–	–	–	14	7
Wedderkopp et al. [45]^a^	Europe	Handball	Organised	1995/1996	126	16–18	Overall	66	45 (68)	19,873	3.3	–	–	–	11	6
							Match	45	–	1925	23.4	–	–	–		
							Training	21	–	17,949	1.2	–	–	–		

*STROBE-SIIS* Strengthening the Reporting of Observational studies in Epidemiology—Sports Injury and Illness Surveillance extension, *NOS* Adapted version of the Newcastle Ottawa Scale

^a^Only control arm of study extracted

### Injury Incidence Rates

**Fig. 2:**
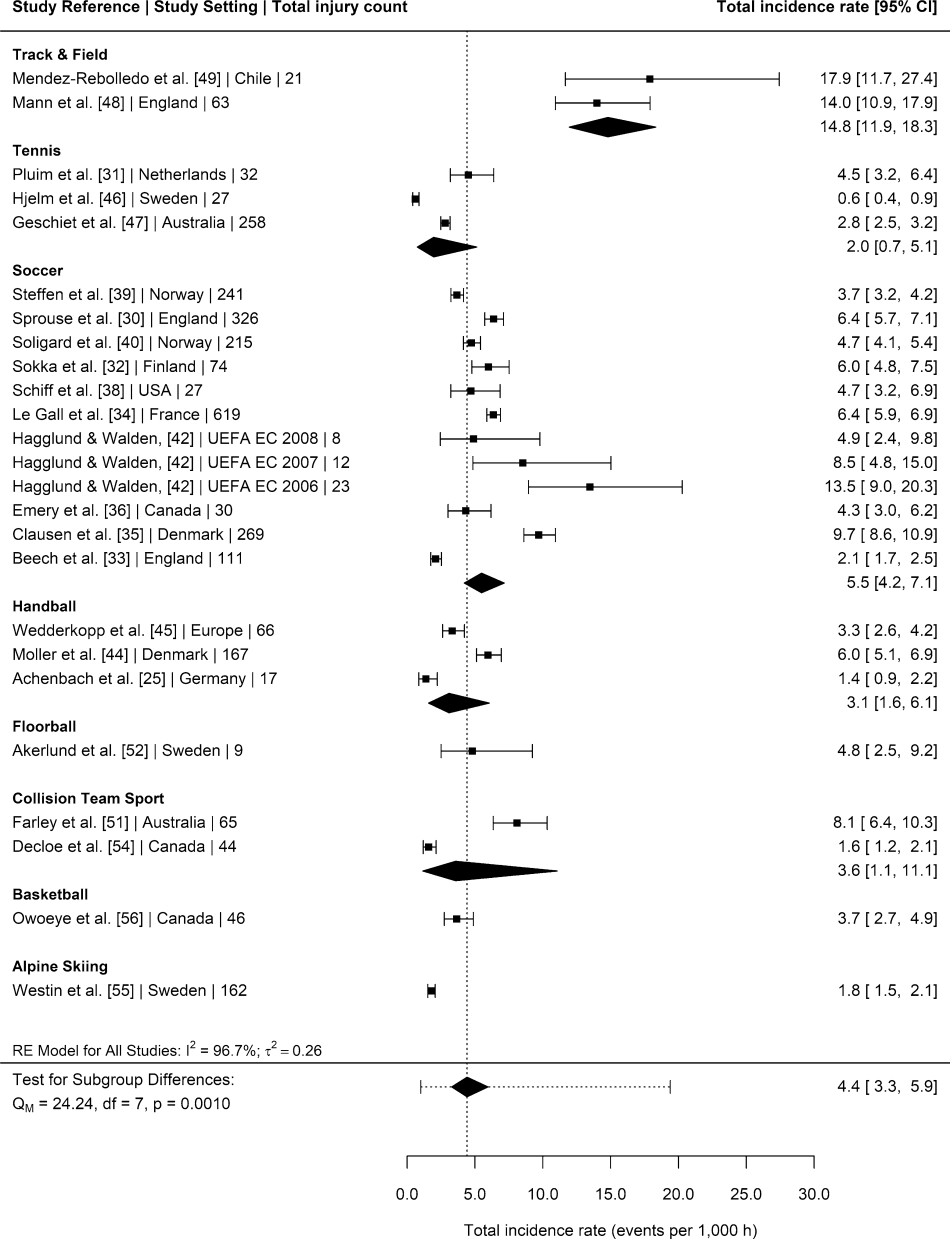
Incidence of total injuries (with 95% confidence intervals) by sport. Study reference, study setting and total number of injuries are provided for each study. The location of the diamond represents the estimated incidence rate and the width reflects the precision of the estimate. The dashed line represents the prediction interval and shows the range of the true effect in 95% of study settings. *RE* random effects

**Fig. 3:**
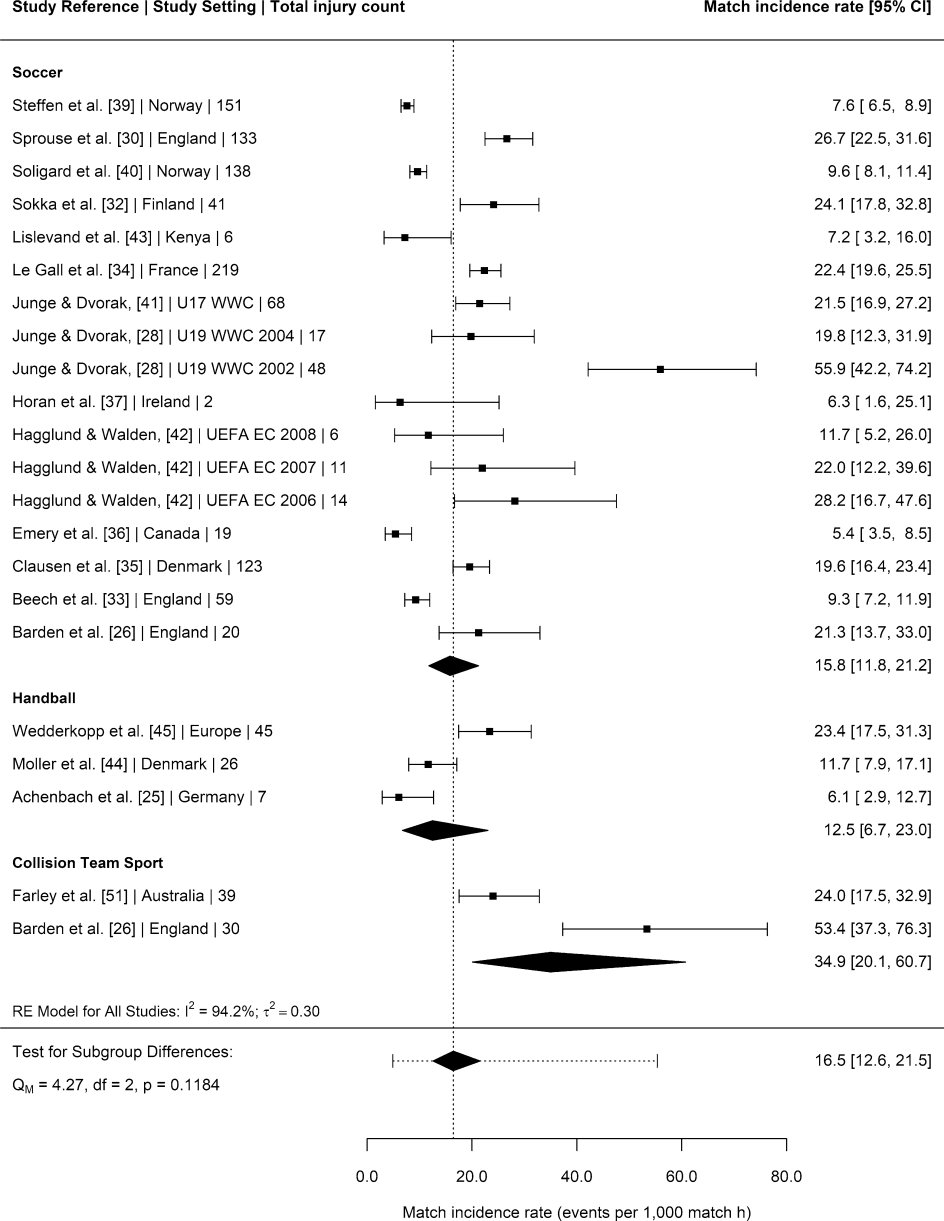
Incidence of match injuries (with 95% confidence intervals) by sport. Study reference, study setting and total number of injuries are provided for each study. The location of the diamond represents the estimated incidence rate and the width reflects the precision of the estimate. The dashed line represents the prediction interval and shows the range of the true effect in 95% of study settings. *RE* random effects

**Fig. 4:**
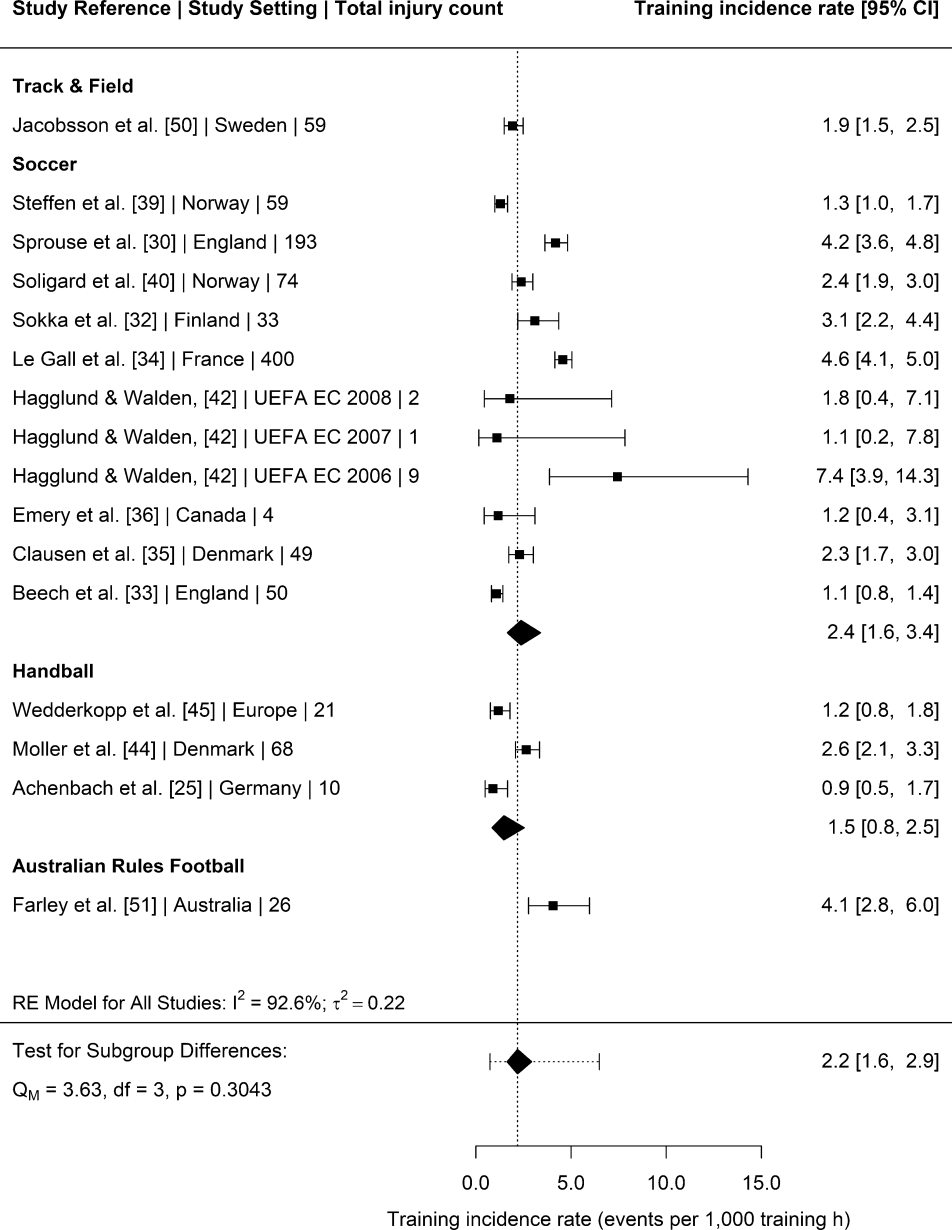
Incidence of training injuries (with 95% confidence intervals) by sport. Study reference, study setting and total number of injuries are provided for each study. The location of the diamond represents the estimated incidence rate and the width reflects the precision of the estimate. The dashed line represents the prediction interval and shows the range of the true effect in 95% of study settings. *RE* random effects

Twenty-three studies [25, 30–36, 38–40, 42–46, 48, 49, 51, 52, 54–56] provided injury surveillance data for all injuries (training and competition/match-play) that could be included in the meta-analysis. The 23 studies reported a total of 2932 injuries amongst youth female athletes exposed to 721,885 exposure hours. Sport was a significant moderator of total injury incidence (*p* = 0.0010) (Fig. 2). Post hoc pairwise comparisons revealed that the pooled incidence rate in track and field athletes was significantly greater than pooled incidences rates of collision-based team sports (*p* = 0.036), handball (*p* = 0.003), soccer (*p* = 0.022) and tennis (*p* = 0.001). Age group, competitive level, recording method, type of competition and study quality were not significant moderators of total injury incidence rates (*p* = 0.5080, *p* = 0.0506, *p* = 0.9668, *p* = 0.6298 and *p* = 0.5227, respectively). Nineteen [25, 26, 28, 30, 32–37, 39–45, 51, 56] studies reported a total of 1222 injuries sustained during matches and 82,834 match hours. Competitive level was a significant moderator of match injury incidence (*p* = 0.0315), with elite youth female athletes having greater pooled injury incidence rates than non-elite athletes (21.9 injury per 1000 h, 95% CI 16–29.8 vs. 12.1 injuries per 1000 h, 95% CI 7.9–18.4, respectively) (Fig. S3, OSM). Sport (Fig. 3), age group, reporting method, competition type and study quality were not significant moderators for match injury incidence (*p* = 0.1184, *p* = 0.3228, *p* = 0.2056, *p* = 0.2952 and *p* = 0.4679, respectively). Fourteen studies [25, 30, 32–36, 39, 40, 42, 44, 45, 50, 51] provided injury surveillance data for training injuries and reported a total of 1053 injuries amongst youth female athletes exposed to 387,948 training hours. Competitive level was a significant moderator of training injury incidence (*p* = 0.0047). Elite youth athletes had greater pooled training incidence rates compared to non-elite athletes (3 injuries per 1000 h, 95% CI 2.1–4.3 vs. 1.5 injuries per 1000 h, 95% CI 1.1–2, respectively) (Fig. S4, OSM). Sport (Fig. 4), age group, reporting method, competition type and study quality were not significant moderators for training injury incidence (*p* = 0.3043, *p* = 0.1029, *p* = 0.1896; *p* = 0.7689 and *p* = 0.7505, respectively).

### Days Missed per Injury

Nine studies [30, 32–34, 36, 42, 47, 48, 51] provided mean days missed for all injuries that could be included in the meta-analysis. The estimated mean days missed per injury was 10 days (95% CI 6–15) with a range of 1 day [47] and 19 days [51] (Fig. S5, OSM). The estimated mean days missed for match injuries from seven studies [26, 30, 32, 33, 36, 41, 51] was 17 days (95% CI 10–24) with a range of 3 days [41] and 45 days [26] (Fig. S6, OSM). The mean days missed for training injuries from five studies [30, 32, 33, 36, 51] was 15 days (95% CI 9–20) with a range of 4 days [32] and 17 days [36] (Fig. S7, OSM).

### Injury Burden Rates

Nine studies [30, 32–34, 36, 42, 47, 48, 51] provided injury surveillance data for overall days lost that could be included in the meta-analysis. These reported a total of 12,469 days lost due to injury amongst youth female athletes exposed to 330,027 exposure hours. This equated to a total burden rate of 46 days per 1000 h of exposure (95% CI 23–92) with range of 3 days [47] and 202 days [42] lost per 1000 h (Fig. S8, OSM). Seven studies [26, 30, 32, 33, 36, 41, 51] reported a total of 7436 days lost due to injury and 22,865 exposure hours equating to a match burden rate of 298 days per 1000 h of exposure (95% CI 137–647) (Fig. S9, OSM). Five studies [30, 32, 33, 36, 51] reported a total of 4625 days lost due to injury and 113,112 exposure hours equating to a training burden rate of 30 days per 1000 h of exposure (95% CI 17–55) (Fig. S10, OSM).

### Meta-Analysed Injury Proportions

**Fig. 5:**
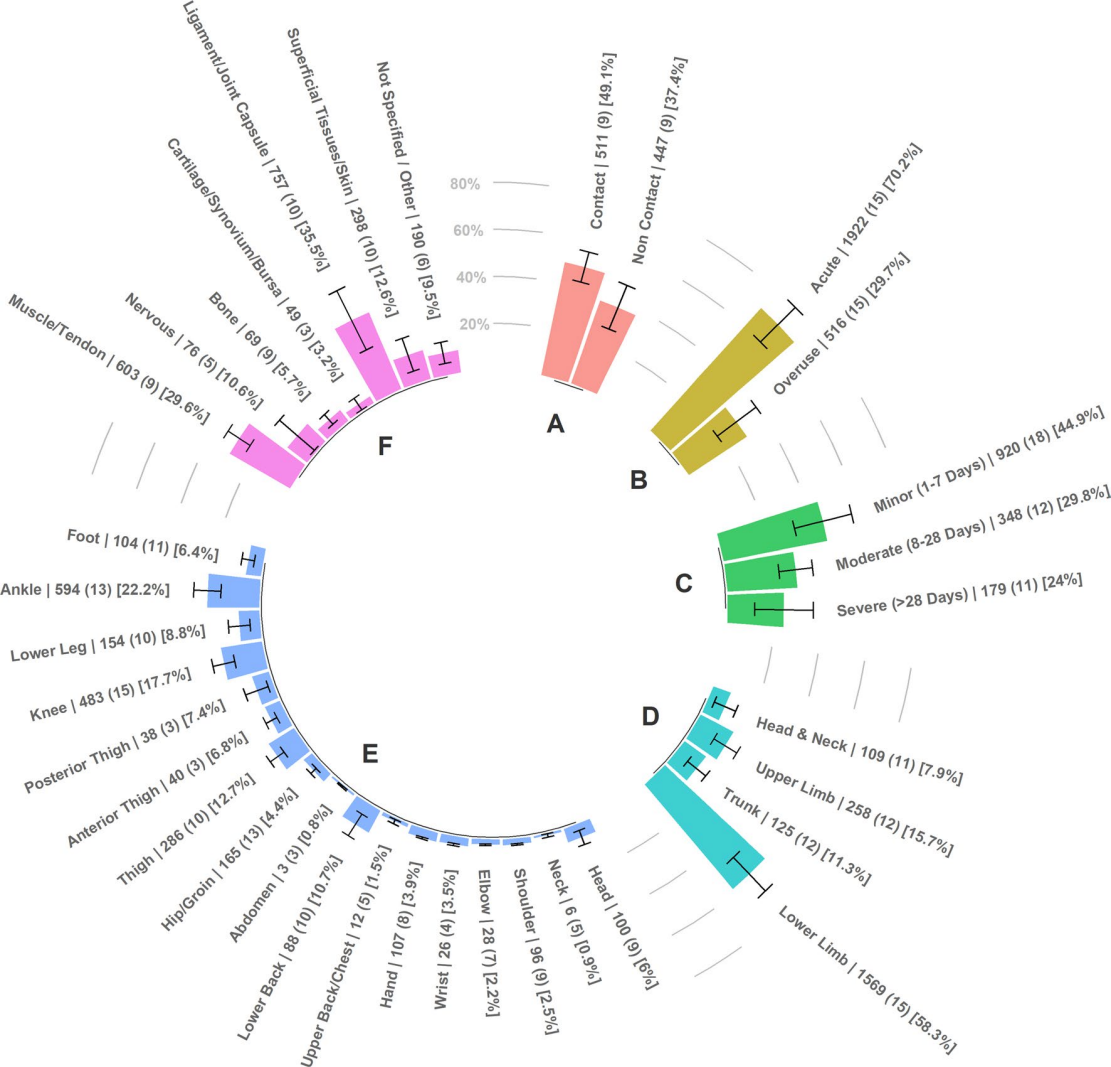
Meta-analysed proportions for injury mechanism (**A**), injury onset (**B**), injury severity time-bins (**C**), injury region (**D**), injury location (**E**) and injury type (**F**). Proportions of each study were combined in the meta-analysis. Error bars represent 95% confidence intervals of estimate proportions. Number: total number of injuries, (): number of studies and []: percentage of total estimated proportion

Meta-analysed proportions of all studies as a function of injury mechanism, onset, severity time-bins, region, location and type are presented in Fig. 5.

#### Injury Prevalence

Twelve studies [26, 27, 29, 31, 34, 38, 42, 45, 46, 50, 53, 55] reported the number of youth female athletes that sustained at least one time-loss injury. Amongst 5393 youth female athletes, 970 sustained one or more time-loss injury resulting in an overall meta-analysed injury prevalence of 39.9% (95% CI 24.8–55.1).

#### Injury Severity Time-Bins

For each injury severity category, a range of from nine to 15 studies [25, 26, 30, 32–34, 36, 39, 41–43, 45, 46, 48, 50, 51, 55, 56] provided data that could be used in the meta-analysis. Minor injuries (1–7 days) were the most common (45%), followed by moderate (8–28 days, 30%) and severe (> 28 days, 24%) severity time-bins (Fig. 5).

#### Injury Mechanism

Nine studies [26, 30, 32, 33, 36, 39, 40, 42, 51] included detail on injury mechanism. Contact mechanisms accounted for most injuries (49%) with non-contact mechanisms accounting for 37% of injuries (Fig. 5).

#### Injury Onset

Thirteen studies [27, 29–31, 33, 34, 39, 40, 42, 45, 46, 50–53] provided data on the onset of injury. Acute injuries accounted for 76% of all injuries, with 25% being overuse in onset (Fig. 5).

#### Injury Location

A range of three to 15 studies [26, 27, 29–35, 39, 40, 45, 46, 48, 50, 53, 55] provided data for each injury region and injury location. The lower limb was the most injured body region (67%), with the ankle (23%), knee (16%) and thigh (13%) being the most prevalent injury locations (Fig. 5).

#### Injury Type

For injury type, a range of three to ten studies [26, 29, 31, 33, 34, 39, 40, 45, 50, 53] included data that could be used in the meta-analysis. Soft-tissue injuries including ligament/joint capsule injuries (42%), muscle/tendon injuries (29%) and superficial injuries (15%) were more common (Fig. 5).

## Discussion

This meta-analysis aimed to compare the injury incidence rates, mean days lost and injury burden rates of youth female athletes participating in different sports. To the authors’ knowledge, this review is the first to meta-analyse mean days lost and injury burden in female youth athletes, two important variables when assessing the risk of injury [11]. The majority of studies included in this review investigated injury in soccer (~ 44%), whilst only single studies were included that assessed injury in popular sports such as rugby union, Australian Rules football, cricket and basketball. One of the main findings of this review was that sport was a significant moderator of overall injury incidence, with track and field presenting a significantly greater pooled injury incidence rate (14.8 injuries per 1000 h, 95% CI 11.9–18.3) than tennis (two injuries per 1000 h, 95% CI 0.7–5.1), soccer (5.5 injuries per 1000 h, 95% CI 4.2–7.1), handball (3.1 injuries per 1000 h, 95% CI 1.6–6.1), and collision-based team sports (3.6 injuries per 1000 h, 95% CI 1.1–11.1). The number of studies included in this review that reported days lost and injury burden data for a given activity (overall *n* = 9, match *n* = 7, training *n* = 5) [24] was insufficient for the analysis of moderators of mean days lost and injury burden rates. The overall pooled mean days lost and injury burden of time loss injuries was 10 days (95% CI 6–15) and 46 days lost per 1000 h (95% CI 23–92), respectively. Age group, injury recording method, competition type or study quality were not significant moderators for any outcome variable. Competitive level was, however, a significant moderator of match and training injury incidence rates, with elite youth female athletes presenting a greater number of injuries per 1000 exposure hours than non-elite youth female athletes (match = 21.9 vs. 12.1 and training = 3 vs. 1.5, respectively). Another aim of this review was to provide the overall injury prevalence for those studies that reported data on the number of athletes that sustained one or more time-loss injuries during the study period (*n* = 12, [26, 27, 29, 31, 34, 38, 42, 45, 46, 50, 53, 55]). The prevalence of a female youth athlete sustaining a time-loss injury was 39.9% (95% CI 24.8–55.1). The majority of time-loss injuries were minor (1–7 days lost) in nature (45%), followed by moderate (30%) and severe (24%) severity time-bins. Generally, most injuries were soft tissue (muscle/tendon = 30% and ligament/joint capsule = 31%) with more severe injuries such as fractures being less common. The lower limb was the most frequently injured body-region, with the ankle (22%), knee (18%) and thigh (13%) being the most commonly injured location site.

### Overall, Match and Training Injury Incidence Rates

To the authors’ knowledge, this is the first review to meta-analyse injury data in youth female athletes from multiple different sport types (e.g., inclusion of team sports, court sports and individual sports). The overall meta-analysed injury incidence rate was 4.4 per 1000 h (95% CI 3.3–5.9), with a significant moderating effect of sport (*p* = 0.0010). As noted in Sect. 5, track and field presented the highest injury incidence rate of 14.8 per 1000 h (95% CI 11.9–18.3). Several contextual and methodological factors may explain this finding. Track and field [48, 50] and endurance sports [49] disciplines require youth athletes to undergo high, consistent and monotonous training intensities, durations and frequencies [50, 58]. The highly repetitive nature of this loading is an injury risk factor and leads to a higher proportion of overuse injuries [59, 60], to which developing youth athletes are particularly susceptible [2]. Indeed, the majority of injuries reported by Mendez-Rebolledo et al. [49] and Mann et al. [48] were overuse in onset (62% and 61% of injuries, respectively). Contrastingly, team sports commonly employ various training modalities of varying intensity such as technical, tactical and physical development training and unlike track and field [50], they are often the attention of injury epidemiological research and subsequent development of injury prevention strategies [61, 62]. Whilst these factors may explain the differences observed for overall injury incidence rates, it is important to highlight the potential influence of methodological discrepancies and limitations of the current review. Firstly, only two studies in track and field [48, 49] were eligible for the current review, and as such, pooled estimates are more susceptible to outliers. Indeed, minimal differences are observed when comparing track and field pooled rates with the two greatest overall rates in soccer (9.7–13.5 injuries/1000 h) [35, 42]. Furthermore, whilst using total pooled incidence rates (combined match and training injuries) allowed for comparison across a greater body of literature and sports, it may provide a false interpretation as combining match and training injuries in team sports can lead to spurious rates with high volumes of training masking high incidence of injury during match-play [63]. Furthermore, not all studies were included in the pooled analysis of injury incidence between sports, and if studies only reporting match incidence rates were included, soccer (15.6 injuries/1000 h) and collision team sports (34.9 injuries/1000 h) were greater than pooled total rates for track and field. Considering the above, the interpretation of comparisons presented in Fig. 2 should be made with caution. This highlights the need for future epidemiological research to characterise injury by activity (matches/competition and training) to help clarify this issue.

Tennis presented the lowest overall pooled injury incidence rate of two injuries per 1000 h (95% CI 0.7–5.1). This agrees with previous conclusions that tennis is a relatively safe and low injury risk sport [64]. The pooled match and training incidence rates for youth female soccer players in the current review are less than those reported in an injury review concerning senior female soccer players [20] (match = 19.2 injuries per 1000 h, 95% CI 16–22.4 and training = 3.5, 95% CI 2.4–4.6). Increasing injury incidence with age is consistent with previous findings in female soccer [33]. Additionally, the overall injury incidence of youth female soccer athletes is comparable to a review by Jones et al. [65] in male youth soccer (5.8 injuries per 1000 h, 95% CI 3.4–10). This is in contrast to the review by Zech et al. [3], which reported an overall incidence rate ratio between female and male youth soccer of 0.75 (95% CI 0.71–0.80), indicating a higher injury risk for males. This is likely explained by methodological differences as unlike Jones et al. [65], the current review included tournament formats that are typically associated with congested fixture periods. Whilst competition structure was not found to be a significant moderator of injury incidence rates, tournament inclusion could have inflated the injury incidence in youth female soccer players as more congested fixture periods have been associated with a more injury risk in soccer [66].

Pooled injury incidences for matches were greater than training (16.5 per 1000 h, 95% CI 12.6–21.5 vs. 2.2 per 1000 h, 95% CI 1.6–2.9, respectively), which is consistent with previous systematic reviews in senior and youth male and female athletes participating in multiple sports [20, 65]. Whilst sport was not a significant moderator of match injury incidence rates, collision team sports presented the greatest pooled injury incidence rates (match = 34.9 injuries per 1000 h) compared to soccer (15.8 injuries per 1000 h) and handball (12.5 injuries per 1000 h) (Fig. 3). Collision-based sports such as rugby union have the highest injury incidence amongst team sports [18]. The repeated contact nature results in many contact injuries being sustained [18], which leads to higher injury incidence rates compared to sports with fewer collision scenarios. Given this finding, more research is required in popular collision team sports, with only one study in rugby union [26] and another in Australian Rules football [51] meeting the criteria of this review. Future research should seek to add to the limited injury data available in these sports particularly if future prophylactic injury reduction strategies are to be developed [7, 67]**.**

### Days Lost and Injury Burden

To the authors’ knowledge, this is the first review to include days lost in youth female sports and the first to assess injury burden in a female sporting context. A pooled estimate of 10 mean days lost per injury (95% CI 6–15) and an injury burden of 46 days lost per 1000 h (95% CI 23–92) was observed overall. Soccer was the only sport to have two or more epidemiological studies (*n* = 6) that reported mean days lost data and injury incidence, which allowed for the calculation of injury burden. The pooled overall mean days lost and injury burden for soccer was 11 days (95% CI 6–16) and 49 days lost per 1000 h (95% CI 28–88), respectively. Consistent with previous findings in male youth [65] and senior female soccer players [20], the pooled mean days lost and injury burden for injuries sustained in matches were greater than for training injuries (15 days, 95% CI 9–22 and 193 days lost per 1000 h, 95% CI 96–378 vs. 14 days, 95% CI 7–22 and 25 injuries per 1000 h, 95% CI 13–47, respectively). Only single studies were available for tennis, distance running, and Australian Rules football, meaning pooled mean days missed and injury burden could not be produced, limiting the ability to draw comparisons between sports. Single studies are not generalisable to a cohort of athletes in a given sport as the epidemiology of injury is highly dependent on contextual factors such as demographic and geographical differences [68], rules and format changes [69], season variability [34] and the organisation's or team's training and competition structure [70]. Visual inspection of mean days lost and injury burden forest plots (Figs. S5 and S8, OSM) show that tennis presented the lowest mean days lost and injury burden, reinforcing the low injury risk of this sport. Distance running presented the second lowest overall mean days lost of 8 days, but when combined with high-incidence rates, presented the second highest injury burden (118 days per 1000 h, 95% CI 108–128) after Australian Rules football (152 injuries per 1000 h, 95% CI 144–161). This follows patterns described by previous research whereby less severe, overuse and non-contact soft-tissue injuries are generally sustained in endurance sports [59, 60], whereas the contact nature of collision team sports such as Australian Rules football and rugby union lends to more severe, traumatic injuries to soft and hard-tissues [71]. For match-play, the pooled mean days lost and injury burden of collision-based teams sports were more than﻿ two- and fivefold greater than for soccer (33 days, 95% CI 9–56 vs. 15 days, 95% CI 9–22 and 1098 days per 1000 h, 95% CI 370–3260 vs. 193 days per 1000 h, 95% CI 98–378, respectively). Again, more serious injuries such as fractures and concussions are more prevalent in collision-based sports such as rugby union and Australian Rules football than soccer [18, 72]. In addition to greater absence from sport, concussion and fractures have been demonstrated to incur greater financial cost for treatment than less severe soft-tissue injuries (i.e., lacerations, muscle strains and contusions) in male youth rugby union [73]. Future research in youth female collision-based team sports should seek to investigate days lost, injury burden and the associated financial cost.

The influence of sport and other moderators on days lost and injury burden data could not be determined due to an insufficient number of studies for this type of analysis [24]. Despite numerous consensus statements advocating the reporting of injury incidence and days lost data for epidemiological studies, only ten [26, 28, 30, 32–34, 36, 47, 48, 51] of the 32 studies included in this review reported information on days lost, injury burden or sufficient information for this variable to be calculated. Interestingly, 19 studies reported injury severity time-bins based on days lost data, but nine failed to report mean or total days lost data. Such additions to reporting would have a big impact on the research base. Furthermore, none of the studies included presented days lost or injury burden data for specific time-loss injury diagnoses, with Gescheit et al. [47] and Mann et al. [48] providing days lost for injury location and Beech et al. [33] providing this information for both injury location and type. Few studies (*n* = 3) [27, 30, 33] also presented this information with reference to the mechanism or circumstances of injury occurrence (e.g., contact, non-contact, running or landing action). The above would help identify potential mechanisms [74] and direct injury management efforts towards those injuries that present the greater burden to youth female athletes in given sports [11]. In contrast to days lost data, approximately 90% of studies (29/32) included in this review reported information on injury incidence or sufficient information for incidence rates to be calculated. Whilst injury incidence is a useful metric, particularly for making comparisons to other datasets due to it being normalised by exposure hours, it only represents the “likelihood” of injury and does not consider the “consequence”. Thus, only reporting injury incidence gives an erroneous and incomplete picture of injury risk [11]. Future research should provide both incidence and days lost data and the cross-product of the two, injury burden (on a total and specific injury level), to allow for a more complete picture of injury risk and, subsequently, allow practitioners working within sport environments to direct efforts towards reducing injuries with the highest burden.

### Injury Characteristics

The overall meta-analysed injury prevalence for youth female athletes was 39.9% (95% CI 24.8–55.1), although these data were obtained from only 12 studies [26, 27, 29, 31, 34, 38, 42, 45, 46, 50, 53, 55]. Almost half of the injuries included in this review were within the minor severity time-bin (1–7 days, 45%), followed by moderate (8–28, 30%) and severe (> 28 days, 24%) time-bins. This pattern is consistent with previous reviews in male and female sport [20, 65]. To maximise the number of studies included when meta-analysing severity time-bin proportions, this review diverged from time-bins suggested by injury consensus statements, and merged slight (1–3 days) and minor (4–7 days) injuries into a single minor time-bin. This was done as ten studies did not categorise injury severity time-bins in line with consensus statements. This lack of consensus of operational definitions limits the accuracy of comparisons between multiple sports and databases. Future research should follow injury reporting guidelines set by consensus statements [10], and in cases where it would be valuable to diverge from these operational definitions such as comparisons to normative data, two sets of severity time-bins should be provided. The meta-analysed proportion of acute injury was threefold that of overuse injury onset (75% vs. 25%, respectively). The high proportion of acute injury could be explained by only investigating time-loss injuries in the current review, which has been found to underestimate overuse injuries in youth sport compared to medical attention and non-time-loss injury assessment [75]. Additionally, the majority of the 13 studies that reported information on injury onset were intermittent team-sports whereas sports that traditionally have high proportions of overuse injuries such a cricket or track and field were fewer and potentially underrepresented in the findings. Consistent with previous reviews assessing injury epidemiology in sport [3, 4, 20, 65], the lower-limb was the most injured body-region (67%). The most common location injured for youth female athletes was the ankle (23%), followed by the knee (16%) and thigh (13%). These locations were consistently reported across sports. Soft-tissue injuries including ligament/joint capsule injuries had the greatest overall meta-analysed proportion (42%) followed by muscle/tendon injuries (29%). Youth female athletes are proposed to be prone to sustaining ligament and joint (non-bone) injuries, mainly around the ankle and knee joints, due to various physiological and biomechanical factors such as neuromuscular control deficits, joint laxity, and increased Q-angles [76–78]. It is important to note that proportions for injury type and location only reflect a relatively small number of studies. Less than half (*n* = 14) of the studies included in the current review provided information on injury location and less than a third (*n* = 10) reported injury types.

### Injury Moderators

Competitive level was a significant moderator of match and training injury incidence rates (*p* = 0.0315 and *p* = 0.0047, respectively), with pooled rates for studies conducted in elite youth female athletes being greater than in studies assessing injury epidemiology in non-elite cohorts (match = 21.9 vs. 12.1 injuries per 1000 h, training = 3 vs. 1.5 injuries per 1000 h, respectively). Unlike non-elite and organised sport participation, a greater proportion of youth athletes in elite sport settings are often specialised, which has been reported to increase injury risk [79, 80]. Furthermore, youth athletes within elite development programmes are subjected to high training exposure volumes and greater match demands and density during periods of rapid growth around peak height velocity, which may predispose them to high injury risk [2, 81, 82]. Decreased resistance of growth cartilage to forces, imbalances in growth and strength, in addition to asynchronous growth of muscle–tendon junctions, growth cartilage and ligaments during rapid growth combined with repetitive loading may explain this heightened risk [2]. However, research concerning growth and biological maturity status with injury has been conducted in male youth athletes [81–83]. Considering sex differences in the timing and tempo of maturation [84], future research concerning growth and biological maturity status in youth female athletes is warranted. It is also important to note that elite environments may have more rigorous capture methods compared to non-elite, which may explain these findings despite injury recording method not being a significant moderator of pooled incidence rates.

None of the other confounding moderators, including age group, injury recording method, competition type, and study quality, had has a significant influence on injury outcomes. Of the 32 studies included in this review, none measured or sought to characterise injury by biological maturity status. It is well established that chronological age is not a good indicator of biological maturity status, which has been shown to impact injury in youth male sport populations [81, 85] and physically active youth females [86]. The assessment of biological maturity status for talent identification, long-term athletic development strategies, and injury risk monitoring purposes is becoming ever present in youth sport [87]. Many studies collect player anthropometrics (height, weight, sitting-height), and several non-invasive biological maturity estimation equations using these data [88, 89] as well as parent heights [90] are readily available and easily employed. Future epidemiological research in youth female sport should collect basic somatic measurements every 3 months [91] to estimate biological maturity  status. There is, however, a need for a standardised approach across the research area on the method of estimation for biological maturity status as maturity-offset [88, 89] and percentage of predicted adult height [90] cannot be used interchangeably [92]. Due to this, it may be pertinent for researchers to collect information required for both methods given they share most somatic variables in their equations. Additionally, given many studies have already collected most of the variables required for estimating biological maturity status, a retrospective analysis of existing data could be feasible to provide the relationship between biological maturity status and injury in youth female athletes. Such research would support practitioners working in youth female sports that currently have to rely on data derived from males concerning injury and biological maturity status, which is erroneous given sex differences in growth and maturation processes [84].

There were no significant differences between different injury recording methods, unlike previous studies comparing self-reported methods to medical and non-medical collection methods [75]. However, these studies concerned medical attention injuries that do not require time-loss and can often be “silent issues” that can be missed by practitioners and/or easily disguised by athletes that continue to participate despite potential injury. Indeed, the number of overuse injuries, often presenting as medical attention injuries, has been found to be significantly different between self-reported and medical practitioners’ collection methods [75]. It is likely the time-loss injury definition applied by this review may reduce incidences as time-loss injuries are obvious and easy to capture. The influence of diagnosis method, which is often different to recording methods [10], on injury diagnoses was not investigated as it is not within the scope of this review. Furthermore, the apparatus (i.e., Oslo Sports Trauma Research Centre questionnaire) used to record injury records was not considered in our analysis.

## Methodological Considerations

### Strengths

To date, this is the first systematic review to examine injury days lost and injury burden in addition to injury incidence in youth female athletes participating in multiple sport settings. The findings of this review describe the injury risk to youth female athletes with the epidemiological data currently accessible for this cohort, allowing for comparisons of injury incidence rates between different sports. Additionally, the effects of potentially confounding moderators such as age, competition type, injury reporting method, and study quality were accounted for in this review. Another strength of the current review is that it highlights current research gaps present in the literature for certain sports. Researchers can use the findings of this review to guide efforts for future injury epidemiological research to sports with a paucity of data. Whilst a strict search strategy was employed in this study to attempt to minimise large between-study heterogeneity, injury recording methods were left open to any method. This was to better reflect many youth sport environments where access to qualified medical personnel is often not possible [93] and third party or self-reported injury data collection methods are employed. Finally, the findings of this review can be used by medical practitioners to benchmark injury risk for given sports included in the review.

### Limitations

This study is not without limitations. Firstly, the systematic search was only performed on the three mentioned databases (PubMed, EBSCO and Cochrane). Whilst manual searching of additional articles from retrieved conference proceedings, posters, review papers and articles was performed, it is possible eligible studies in other databases and located in other sources such as websites and stakeholder reports were missed during the search process. Secondly, this study focused on the epidemiology of injury and not the long-term consequences of injury such as forced retirements, reduction in physical activity, long-term health consequences and psychosocial factors**.** These are potentially important metrics for governing bodies in sports and health services worldwide to obtain a full picture of the consequences of sport injuries. However, many follow-up periods of studies are not long enough in duration and do not allow for factors outside of those directly impacting sports participation to be assessed. Future research and reviews should seek to investigate these factors. Thirdly, an inherent limitation of this review is the paucity of available epidemiological research across a broad range of youth female sports. Low density of research available in sports other than soccer means estimates cannot be calculated, which limits comparison between sports, and in cases such as rugby union and Australian Rules football, steps were taken to merge these into ‘collision-based team sports’ to allow for comparisons. Additionally, analysis comparing mean days lost and injury burden could not be performed due to the limited number of studies that present days lost and injury burden metrics. However, this limitation is not unique to the present review as a recent review of elite female soccer players [20] was unable to attain injury burden from available research. Fourthly, the time-loss injury definition screening criteria employed may have led to an underestimation of the number of overuse injuries sustained in youth cohorts [75], which often do not present time-loss but have significant health and performance consequences [2]**.** However, whilst broad injury definitions including all complaint incidents allow for more injuries to be captured [75, 94], there is the potential to collect injuries with less reliability [95], less importance, and less interest [65]. By employing an objective time-loss definition, it is easier to align studies and remove the subjectivity about which health issue is “worth” recording or not between practitioners. Moreover, one of the main aims of this review was to investigate days lost data and injury burden, and thus a focus on time-loss injuries was warranted.

### Future Perspectives

The information in this review demonstrates the injury risk for youth female athletes and the need for evidence-based injury prevention programmes to reduce the negative effects of injury. This review also highlights the relative paucity of epidemiological studies available across a broad range of youth female sports in addition to the lack of studies assessing days lost due to injury and injury burden. A further challenge is the low density of research available in some sports, with soccer being the only one to have more than four studies available reporting injury incidence. To overcome the challenges presented by this review, routine multi-year injury surveillance following recommendations of current consensus statements needs to be employed in all youth female sport settings. Such data are critical for increasing the breadth and depth of the epidemiological database in youth female athletes, which is essential if evidenced-based injury prevention strategies are to be developed.

## Conclusion

The current review illustrates that a limited database of injury surveillance studies is available across youth female sports (*n* = 32) despite a broad inclusion criterion. Outside of soccer, little research density is evidenced with single studies available in popular team sports such as Australian Rules football and rugby union. Whilst incidence or sufficient data for its calculation were presented by 90% of studies included, only ten reported information on days lost or injury burden. This number was further reduced to three for presenting this information on an injury location, type or mechanism level. As a result of insufficient study numbers, analysis and pooled estimates for mean days lost and injury burden could only be completed for soccer. This highlights the need for future research to report days lost data alongside injury number and exposure at both an activity (competition and training) and specific injury level (location, type, diagnosis and mechanism) so burden can be calculated and the full risk of injury to youth female athletes can be identified. This will support targeted interventions for those injuries causing the greatest burden in youth female sport.

### Supplementary Information

Below is the link to the electronic supplementary material.

Supplementary file1 (PDF 179 KB)

Supplementary file2 (PDF 306 KB)

Supplementary file3 (PDF 302 KB)

Supplementary file4 (PDF 304 KB)

Supplementary file5 (PDF 178 KB)

Supplementary file6 (PDF 171 KB)

Supplementary file7 (PDF 129 KB)

Supplementary file8 (PDF 208 KB)

Supplementary file9 (PDF 193 KB)

Supplementary file10 (PDF 170 KB)

Supplementary file11 (DOCX 47 KB)

## Declarations

### Availability of data and material

Via the Open Science Framework at: https://osf.io/53t4q/

### Code availability

Via the Open Science Framework at: https://osf.io/53t4q/

### PROSPERO

PROSPERO registration number: CRD42021290401. Accessed at www.crd.york.ac.uk/prospero/display_record.php?RecordID=290401.
