# Supplementary material for: Injury Profile in Youth Female Athletes: A Systematic Review and Meta-Analysis
Source: Sports Med. 2024 Jan 24;54(5):1207–30. doi: 10.1007/s40279-023-01988-w (PMC11127887; doi:10.1007/s40279-023-01988-w)
Supplement: Supplementary file 8 — Supplementary file8 (PDF 208 KB) [file 40279_2023_1988_MOESM8_ESM.pdf]

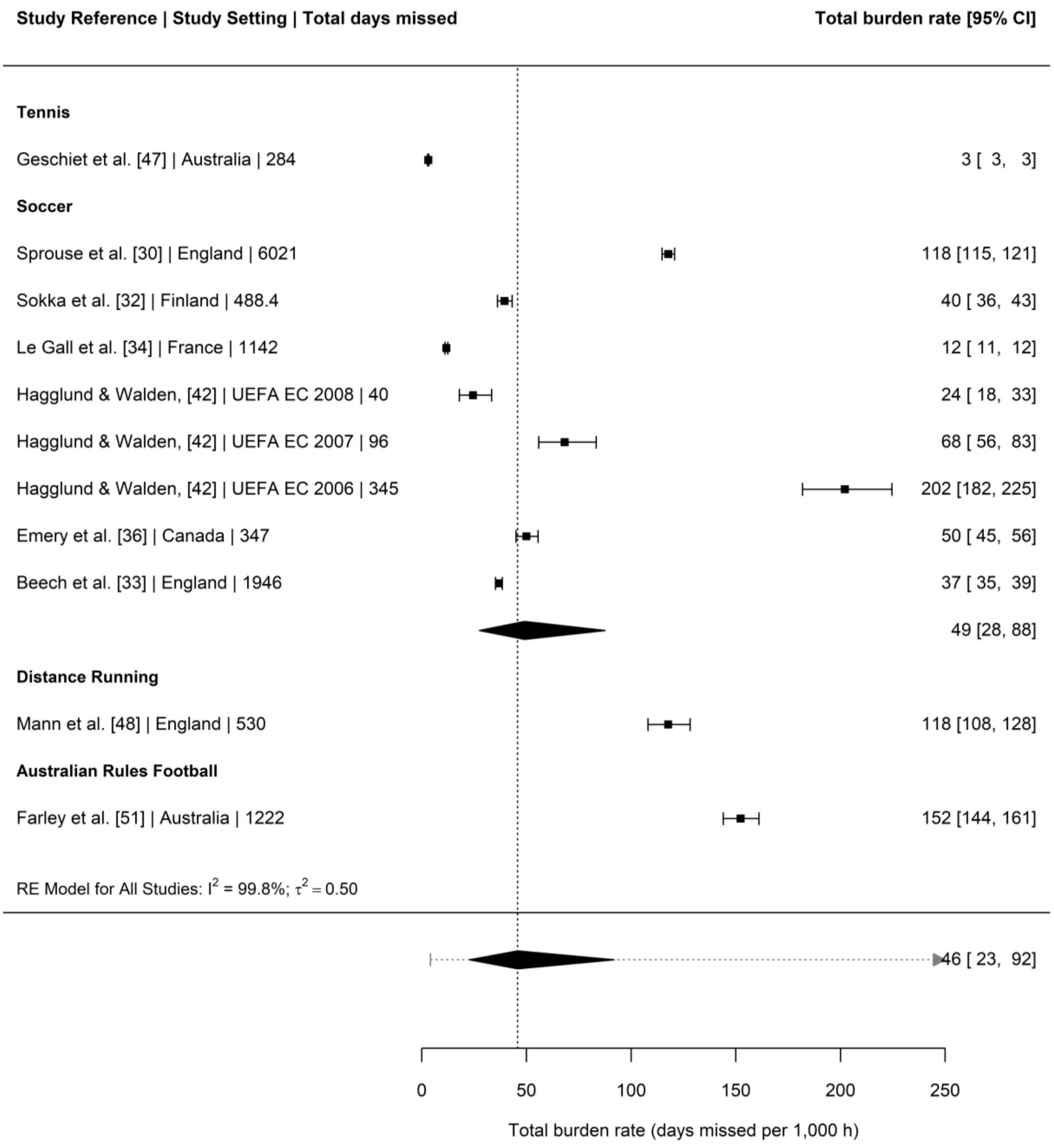

Fig. S8 Burden of injuries (with 95% confidence intervals) by sport. Study reference, study setting, and total number of injuries are provided for each study. The location of the diamond representing the estimated injury burden and the width reflects the precision of the estimate. The dashed line represents the prediction interval and shows the range of the true effect in 95% of study settings. RE: Random Effects

Title: Injury Profile in Youth Female Athletes: Systematic Review and Meta-Analysis

Journal Name: Sports Medicine

Authors: Jake Beech[1,2], Ben Jones[1,3,4,5,6], Thomas Hughes[1], Stacey Emmonds[1,2]

Affiliations:

[1] Carnegie School of Sport, Leeds Beckett University, Leeds, UK

[2] The Football Association, Burton-Upon-Trent, UK

[3] England Performance Unit, The Rugby Football League, Leeds, UK
[4] Leeds Rhinos Rugby League club, Leeds, UK
[5] Division of Exercise Science and Sports Medicine, Department of Human Biology, Faculty of Health Sciences, the University of Cape Town and the Sports Science Institute of South Africa, Cape Town, South Africa

[6] School of Science and Technology, University of New England, Armidale, NSW, Australia.

Corresponding Author:

Jake Beech

E: j.e.beech@leedsbeckett.ac.uk
