# Supplementary material for: Injury Profile in Youth Female Athletes: A Systematic Review and Meta-Analysis
Source: Sports Med. 2024 Jan 24;54(5):1207–30. doi: 10.1007/s40279-023-01988-w (PMC11127887; doi:10.1007/s40279-023-01988-w)
Supplement: Supplementary file 11 — Supplementary file11 (DOCX 47 KB) [file 40279_2023_1988_MOESM11_ESM.docx]

**Table S1. Concepts and search terms**

|  | **Concept 1** | **Concept 2** | **Concept 3** | **Concept 4** | **Concept 5** |
| --- | --- | --- | --- | --- | --- |
| **Key concepts** | **Injury type** | **Study design** | **Sport** | **Sex** | **Level/age** |
| **Free text terms / natural language terms – screen in tables and abstracts (tiab) and key words**  (synonyms, UK/US terminology, medical/laymen’s terms, acronyms/abbreviations, drug brands, more narrow search terms)  *Consider: phrase searching, proximity operators, truncation, wildcards, field qualification (e.g. textword)* | Injury, ACL, anterior cruciate ligament, muscle, ligament, tendon, sprains, strains, tears, contusion, haematoma, dislocation, concussion, fracture, rupture, lower extremity, knee, ankle, hip, hamstring, quadriceps, time loss injury, body site | Incidence, epidemiology, prevalence, severity, burden, aetiology, surveillance, survey, audit, injury patterns, prospective study | Football, soccer, futsal, basketball, handball, netball, volleyball, rugby, rugby union, rugby league, Australian rules football, hockey, lacrosse, hurling, softball, cricket, golf, badminton, tennis, athletics, track and field, martial arts, gymnastics, running, cross country, gymnasts, squash, dance, table tennis. Race walking, swimming, diving, rowing, skiing, skiers, American football, Sprinters | Female, girl, girls, gender, sex | Elite, youth, adolescent, child, academy, high-level, talented, scholarship |
| **Controlled vocabulary** **terms / Subject terms**  (MeSH terms) – depending on database  *Consider: explode, major headings, subheadings* | Wounds and injuries, anterior cruciate ligament, muscles, ligaments, Lower extremity, Head, Torso, upper extremity | Incidence, epidemiology | Sports, football, soccer, racket sports, team sports, track and field, martial arts, water sports, snow sports | Female |  |

**Search Strategy**

**PUBMED –**

#1 Injury type

(“wounds and injuries”[MeSH] OR “injury”[tiab] OR “anterior cruciate ligament”[MeSH] OR “ACL”[tiab] OR “muscles”[MeSH] OR “muscles”[tiab] OR “ligaments”[MeSH] OR “ligaments”[tiab] OR “tendon”[tiab] OR “sprains”[tiab] OR “strains”[tiab] OR “tears”[tiab] OR “contusion”[tiab] OR “haematoma”[tiab] OR “dislocation”[tiab] OR “concussion”[tiab] OR “fracture”[tiab] OR “rupture”[tiab] OR “lower extremity”[MeSH] OR “lower extremity”[tiab] OR “head”[MeSH] OR “torso”[MeSH] OR “upper extremity” [MeSH])

#2 Study design

(“incidence”[MeSH] OR “incidence”[tiab] OR “epidemiology”[MeSH] OR “epidemiology”[tiab] OR “prevalence”[tiab] OR “severity”[tiab] OR “burden”[tiab] OR “aetiology”[tiab] OR “surveillance”[tiab] OR “survey”[tiab] OR “audit”[tiab] OR “injury patterns”[tiab] OR “prospective study”[tiab])

#3 Sport

(“sports”[MeSH] OR “sport”[tiab] OR “team sports”[MeSH] OR “team sports”[tiab] OR “football”[MeSH] OR “football”[tiab] OR “soccer”[MeSH] OR “soccer”[tiab] OR “futsal”[tiab] OR “basketball”[tiab] OR “handball”[tiab] OR “netball”[tiab] OR “volleyball”[tiab] OR “rugby”[tiab] OR “rugby union”[tiab] OR “rugby league”[tiab] OR “australian rules football”[tiab] OR “hockey” OR “field hockey”[tiab] OR “lacrosse”[tiab] OR “hurling”[tiab] OR “softball”[tiab] or “cricket”[tiab] OR “racquet sports”[MeSH] OR “badminton”[tiab] OR “tennis”[tiab] OR “squash”[tiab] OR “table tennis”[tiab] OR “track and field”[MeSH] OR “athletics”[tiab] OR “sprinters”[tiab] OR “running”[tiab] OR “cross country”[tiab] OR “race walking”[tiab] OR “gymnastics”[tiab] OR “dance”[tiab] OR “martial arts”[MeSH] OR “snow sports”[MeSH] OR “skiing”[tiab] OR “skiers”[tiab] OR “figure skating”[tiab] OR “water sports”[MeSH] OR “swimming”[tiab] OR “diving”[tiab] OR “rowing”[tiab] OR “weightlifting”[tiab])

#4 Sex

(“female”[MeSH] OR “female”[tiab] OR “girl”[tiab] OR “girls”[tiab] OR “gender”[tiab])

#5 Population/age

(“elite”[tiab] OR “youth”[tiab] OR “adolescent”[tiab] OR “child”[tiab] OR “academy”[tiab] OR “high-level”[tiab] OR “talented”[tiab] OR “scholarship”[tiab])

**EBSCOE** (SportDiscus with Full Text, MEDLINE, APA PsycIinfo, CINAHL, Academic Search Complete)

#1 Injury type

((MH “wounds and injuries”+) OR (TI “injury” OR AB “injury”) OR (MH “anterior cruciate ligament”+) OR (TI “anterior cruciate ligament” OR AB “anterior cruciate ligament”) OR (TI “ACL” OR AB “ACL”) OR (MH “muscles”+) OR (TI “muscles” OR AB “muscles”) OR (MH “ligaments”+) OR (TI “ligaments” OR AB “ligaments”) OR (TI “tendon” OR AB “tendon”) OR (TI “sprains” OR AB “strains”) OR (TI “tears” OR AB “tears”) OR (TI “contusion” OR AB “contusion”) OR (TI “haematoma” OR AB “haematoma”) OR (TI “dislocation” OR AB “dislocation”) OR (TI “concussion” OR AB “concussion”) OR (TI “fracture” OR AB “fracture) OR (TI “rupture” OR AB “rupture”) OR (MH “lower extremity”+) OR (TI “lower extremity” OR AB “lower extremity”) OR (MH “head”+) OR (MH “torso”+) OR (MH “upper extremity”+))

#2 Study design

((MH “incidence”+) OR (TI “incidence” OR AB “incidence”) OR (MH “epidemiology”+) OR (TI “epidemiology” OR AB “epidemiology”) OR (TI “prevalence” OR AB “prevalence”) OR (TI “severity” OR AB “severity”) OR (TI “burden” OR AB “burden”) OR (TI “aetiology” OR AB “aetiology”) OR (TI “surveillance” OR AB “surveillance”) OR (TI “survey” OR AB “survey”) OR (TI “audit” OR AB “audit”) OR (TI “injury patterns” OR AB “injury patterns”) OR (TI “prospective study” OR AB “prospective study”))

#3 Sport

((MH “sports”+) OR (TI “sport” OR AB “sport) OR (MH “team sports”+) OR (TI “team sports” OR AB “team sports”) OR (MH “football”+) OR (TI “football” OR AB “football”) OR (MH “soccer”+) OR (TI “soccer” OR AB “soccer”) OR (TI “futsal” OR AB “futsal”) OR (TI “basketball” OR AB “basketball”) OR (TI “handball” OR AB “handball”) OR (TI “netball” OR AB “netball”) OR (TI “volleyball” OR AB “volleyball”) OR (TI “rugby” OR AB “rugby”) OR (TI “rugby union” OR AB “rugby union”) OR (TI “rugby league” OR AB “rugby league”) OR (TI “australian rules football” OR AB “Australian rules football”) OR (TI “hockey” OR AB “hockey”) OR (TI “field hockey” OR AB “field hockey) OR (TI “lacrosse” OR AB “lacrosse”) OR (TI “hurling” OR AB “hurling”) OR (TI “softball” OR AB “softball”) OR (TI “cricket” OR AB “cricket”) OR (MH “racquet sports”+) OR (TI “badminton” OR AB “badminton”) OR (TI “tennis” OR AB “tennis”) OR (TI “squash” OR AB “squash”) OR (TI “table tennis” OR AB “table tennis”) OR (MH “track and field”+) OR (TI “athletics” OR AB “athletics”) OR (TI “sprinters” OR AB “sprinters”) OR (TI “running” OR AB “running”) OR (TI “cross country” OR AB “cross country”) OR (TI “race walking” OR AB “race walking”) OR (TI “gymnastics” OR AB “gymnastics”) OR (TI “dance” OR AB “dance”) OR (MH “martial arts”+) OR (MH “water sports”+) OR (TI “swimming” OR AB “swimming”) OR (TI “diving” OR AB “diving”) OR (TI “rowing” OR AB “rowing”) OR (TI “weightlifting” OR AB “weightlifting”) OR (MH “snow sports”+) OR (TI “skiing” OR AB “skiing”) OR (TI “skiers” OR AB “skiers”) OR (TI “figure skating” OR AB “figure skating”))

#4 Sex

((MH “female”+) OR (TI “female” OR AB “female”) OR (TI “girl” OR AB “girl”) OR (TI “girls” OR AB “girls”) OR (TI “gender” OR AB “gender”))

#5 Level/age

((TI “elite” OR AB “elite”) OR (TI “youth” OR AB “youth”) OR (TI “adolescent” OR AB “adolescent”) OR (TI “child” OR AB “child”) OR (TI “academy” OR AB “academy”) OR (TI “high-level” OR AB “high-level”) OR (TI “talented” OR AB “talented”) OR (TI “scholarship” OR AB “scholarship”))

**Cochrane-**

#1 Injury type

(“wounds and injuries” or “injury” or “anterior cruciate ligament” or “ACL” or “muscles” or “ligaments” or “tendon” or “sprains” or “strains” or “tears” or “contusion” or “haematoma” or “dislocation” or “concussion” or “fracture” or “rupture” or “lower extremity” or “lower extremity” or “head” or “torso” or “upper extremity”)

ti,ab,kw

#2 Study design

(“incidence” or “epidemiology” or “prevalence” or “severity” or “burden” or “aetiology” or “surveillance” or “survey” or “audit” or “injury patterns” or “prospective study”)

ti,ab,kw

#3 Sport

(“sports” or “sport” or “team sports” or “football” or “soccer” or “futsal” or “basketball” or “handball” or “netball” or “volleyball” or “rugby” or “rugby union” or “rugby league” or “australian rules football” or “hockey” or “field hockey” or “lacrosse” or “hurling” or “softball” or “cricket” or “racquet sports” or “badminton” or “tennis” or “squash” or “table tennis” or “track and field” or “athletics” or “sprinters” or “running” or “cross country” or “race walking” or “gymnastics” or “dance” or “martial arts” or “water sports” or “swimming” or “diving” or “rowing” or “weightlifting” or “snow sports” or “skiing” or “skiers” or “figure skating”)

ti,ab,kw

#4 Sex

(“female” or “girl” or “girls” or “gender”)

ti,ab,kw

#5 Level/age

(“elite” or “youth” or “adolescent” or “child” or “academy” or “high-level” or “talented” or “scholarship”)

ti,ab,kw

Table S2: Individual Study Rating For STROBE-SIIS Extension

|  | **STROBE-SIIS Extension Item** | | | | | | | | | | | | | | | | | | | | | | | |  | |
| --- | --- | --- | --- | --- | --- | --- | --- | --- | --- | --- | --- | --- | --- | --- | --- | --- | --- | --- | --- | --- | --- | --- | --- | --- | --- | --- |
| **Author/study** | **1** | **2** | **3** | **4** | **5** | **6** | **7** | **8** | **9** | **10** | **11** | **12** | **13** | **14** | **15** | **16** | **17** | **18** | **19** | **20** | **21** | **22** | **23** | **Total** | |  |
| Achenbach et al. [25] | 0 | 1 | 0 | 0 | 0 | 1 | 1 | 0 | 0 | 1 | 0 | 0 | 1 | 1 | 1 | 0 | 1 | 1 | 1 | 1 | 1 | 1 | 0 | 13 | |  |
| Akerlund et al. [52] | 1 | 1 | 1 | 0 | 1 | 1 | 1 | 1 | 0 | 1 | 0 | 0 | 1 | 1 | 1 | 1 | 0 | 1 | 1 | 1 | 1 | 1 | 0 | 17 | |  |
| Barden et al. [26] | 0 | 1 | 0 | 0 | 1 | 1 | 1 | 0 | 0 | 0 | 0 | 0 | 0 | 1 | 1 | 1 | 1 | 1 | 1 | 1 | 1 | 1 | 0 | 13 | |  |
| Beech et al. [33] | 1 | 1 | 0 | 0 | 1 | 1 | 1 | 0 | 0 | 0 | 1 | 1 | 0 | 1 | 1 | 1 | 1 | 1 | 1 | 1 | 1 | 1 | 0 | 16 | |  |
| Clausen et al. [35] | 1 | 1 | 0 | 0 | 1 | 1 | 1 | 0 | 0 | 0 | 0 | 1 | 1 | 1 | 0 | 1 | 0 | 1 | 0 | 1 | 1 | 1 | 1 | 14 | |  |
| Decloe et al. [54] | 1 | 1 | 0 | 0 | 1 | 1 | 1 | 1 | 0 | 1 | 0 | 1 | 0 | 1 | 1 | 1 | 0 | 1 | 1 | 1 | 1 | 1 | 0 | 16 | |  |
| Emery et al. [36] | 1 | 1 | 0 | 0 | 0 | 1 | 1 | 1 | 1 | 0 | 0 | 0 | 1 | 1 | 1 | 1 | 1 | 1 | 1 | 1 | 1 | 0 | 0 | 15 | |  |
| Farley et al. [51] | 1 | 1 | 0 | 1 | 1 | 1 | 1 | 0 | 0 | 1 | 1 | 1 | 1 | 1 | 1 | 1 | 1 | 1 | 1 | 1 | 1 | 1 | 0 | 19 | |  |
| Geschiet et al. [47] | 1 | 1 | 0 | 1 | 1 | 1 | 1 | 0 | 0 | 0 | 1 | 1 | 0 | 1 | 1 | 1 | 0 | 1 | 1 | 1 | 1 | 1 | 1 | 17 | |  |
| Goggins et al. [27] | 1 | 1 | 0 | 1 | 1 | 1 | 1 | 0 | 0 | 0 | 0 | 1 | 0 | 1 | 1 | 1 | 0 | 1 | 1 | 1 | 1 | 1 | 1 | 16 | |  |
| Hagglund & Walden, [42] | 1 | 1 | 0 | 0 | 1 | 1 | 1 | 0 | 0 | 0 | 0 | 0 | 1 | 1 | 1 | 1 | 1 | 1 | 1 | 1 | 1 | 1 | 0 | 15 | |  |
| Hjelm et al. [46] | 1 | 1 | 0 | 1 | 1 | 1 | 1 | 0 | 0 | 0 | 0 | 0 | 1 | 1 | 1 | 1 | 1 | 1 | 1 | 1 | 0 | 0 | 0 | 14 | |  |
| Horan et al. [37] | 1 | 1 | 0 | 0 | 1 | 1 | 1 | 0 | 0 | 0 | 0 | 0 | 1 | 1 | 1 | 1 | 1 | 1 | 1 | 1 | 1 | 1 | 0 | 15 | |  |
| Jacobsson et al. [50] | 1 | 1 | 0 | 1 | 1 | 1 | 1 | 1 | 0 | 1 | 0 | 0 | 1 | 1 | 1 | 0 | 1 | 1 | 1 | 1 | 1 | 1 | 0 | 17 | |  |
| Junge & Dvorak, [28] | 1 | 1 | 0 | 0 | 1 | 1 | 1 | 1 | 0 | 0 | 0 | 1 | 0 | 1 | 1 | 1 | 1 | 1 | 0 | 1 | 1 | 1 | 0 | 15 | |  |
| Junge & Dvorak, [41] | 1 | 1 | 0 | 0 | 1 | 1 | 1 | 0 | 0 | 0 | 0 | 0 | 0 | 1 | 0 | 1 | 1 | 1 | 1 | 1 | 1 | 1 | 0 | 13 | |  |
| Le Gall et al. [34] | 1 | 1 | 0 | 0 | 1 | 1 | 1 | 1 | 0 | 0 | 0 | 1 | 0 | 1 | 0 | 1 | 1 | 1 | 1 | 1 | 1 | 0 | 0 | 14 | |  |
| Lislevand et al. [43] | 1 | 1 | 0 | 1 | 1 | 1 | 1 | 0 | 0 | 1 | 0 | 0 | 0 | 1 | 0 | 1 | 1 | 1 | 0 | 1 | 1 | 1 | 0 | 14 | |  |
| Mann et al. [48] | 1 | 1 | 0 | 0 | 1 | 1 | 1 | 0 | 1 | 1 | 0 | 1 | 1 | 1 | 1 | 1 | 0 | 1 | 1 | 1 | 1 | 1 | 0 | 17 | |  |
| McGuine et al. [29] | 1 | 1 | 0 | 0 | 0 | 1 | 1 | 0 | 0 | 1 | 0 | 0 | 1 | 1 | 1 | 1 | 1 | 1 | 1 | 1 | 1 | 0 | 0 | 14 | |  |
| Mendez-Rebolledo et al. [49] | 1 | 1 | 0 | 0 | 1 | 1 | 1 | 0 | 0 | 1 | 0 | 0 | 0 | 0 | 0 | 1 | 0 | 1 | 1 | 1 | 1 | 0 | 1 | 12 | |  |
| Moller et al. [44] | 1 | 1 | 0 | 0 | 1 | 1 | 1 | 0 | 0 | 0 | 0 | 0 | 1 | 1 | 1 | 1 | 1 | 1 | 1 | 1 | 1 | 0 | 0 | 14 | |  |
| Owoeye et al. [56] | 1 | 1 | 0 | 0 | 1 | 1 | 1 | 1 | 0 | 1 | 0 | 0 | 1 | 1 | 1 | 1 | 1 | 1 | 1 | 1 | 1 | 1 | 0 | 17 | |  |
| Pluim et al. [31] | 1 | 1 | 0 | 1 | 1 | 1 | 1 | 1 | 0 | 0 | 0 | 0 | 1 | 1 | 1 | 1 | 0 | 1 | 1 | 1 | 1 | 0 | 0 | 15 | |  |
| Schiff et al. [38] | 0 | 1 | 0 | 0 | 1 | 1 | 1 | 0 | 1 | 0 | 0 | 1 | 1 | 1 | 0 | 1 | 0 | 1 | 1 | 1 | 1 | 0 | 0 | 13 | |  |
| Sokka et al. [32] | 1 | 1 | 0 | 0 | 1 | 1 | 1 | 1 | 0 | 0 | 0 | 1 | 1 | 1 | 1 | 1 | 0 | 1 | 1 | 1 | 1 | 1 | 0 | 16 | |  |
| Soligard et al. [40] | 1 | 1 | 1 | 0 | 1 | 1 | 1 | 1 | 0 | 1 | 0 | 1 | 1 | 0 | 1 | 1 | 1 | 1 | 1 | 1 | 1 | 1 | 0 | 18 | |  |
| Sprouse et al. [30] | 1 | 1 | 0 | 1 | 1 | 1 | 1 | 0 | 0 | 0 | 0 | 0 | 0 | 1 | 0 | 1 | 1 | 1 | 1 | 1 | 1 | 1 | 1 | 15 | |  |
| Steffen et al. [39] | 1 | 1 | 0 | 0 | 1 | 1 | 1 | 0 | 0 | 1 | 0 | 0 | 1 | 1 | 1 | 1 | 1 | 1 | 1 | 1 | 1 | 1 | 0 | 16 | |  |
| Watson et al. [53] | 1 | 1 | 0 | 0 | 1 | 1 | 1 | 0 | 0 | 0 | 0 | 0 | 1 | 1 | 1 | 0 | 1 | 1 | 1 | 1 | 1 | 1 | 0 | 14 | |  |
| Wedderkopp et al. [45] | 1 | 1 | 0 | 0 | 1 | 1 | 1 | 0 | 0 | 0 | 0 | 1 | 0 | 1 | 1 | 0 | 0 | 1 | 0 | 1 | 0 | 1 | 0 | 11 | |  |
| Westin et al. [55] | 1 | 1 | 0 | 1 | 1 | 1 | 1 | 0 | 0 | 0 | 0 | 0 | 1 | 1 | 1 | 1 | 0 | 1 | 1 | 1 | 0 | 1 | 0 | 14 | |  |
|  |  |  |  |  |  |  |  |  |  |  |  |  |  |  |  |  |  |  |  |  |  |  | **Mean** | 15 | |  |
|  |  |  |  |  |  |  |  |  |  |  |  |  |  |  |  |  |  |  |  |  |  |  | **SD** | 2 | |  |

Table S3: Individual study rating for NOS for assessing risk of bias

|  | **NOS Criteria** | | | | | | | | |
| --- | --- | --- | --- | --- | --- | --- | --- | --- | --- |
| **Author/study** | **1** | **2** | **3** | **4** | **5** | **6** | **7** | **8** | **Total** |
| Achenbach et al. [25] |  | ***** | ***** | ***** |  | ***** | ***** | ***** | 6 |
| Akerlund et al. [52] | ***** | ***** | ***** | ***** |  | ***** | ***** | ***** | 7 |
| Barden et al. [26] | ***** | ***** |  | ***** |  | ***** | ***** |  | 5 |
| Beech et al. [33] | ***** | ***** | ***** | ***** |  | ***** | ***** |  | 6 |
| Clausen et al. [35] | ***** | ***** | ***** | ***** |  | ***** | ***** | ***** | 7 |
| Decloe et al. [54] | ***** | ***** | ***** | ***** | ***** | ***** | ***** |  | 7 |
| Emery et al. [36] | ***** | ***** | ***** | ***** |  | ***** | ***** | ***** | 7 |
| Farley et al. [51] | ***** | ***** | ***** | ***** |  | ***** | ***** |  | 7 |
| Geschiet et al. [47] | ***** | ***** | ***** | ***** |  | ***** | ***** |  | 6 |
| Goggins et al. [27] | ***** | ***** | ***** | ***** |  | ***** | ***** | ***** | 6 |
| Hagglund & Walden, [42] | ***** |  | ***** | ***** |  | ***** | ***** | ***** | 7 |
| Hjelm et al. [46] | ***** |  | ***** | ***** |  | ***** | ***** | ***** | 7 |
| Horan et al. [37] | ***** | ***** |  | ***** |  | ***** | ***** |  | 7 |
| Jacobsson et al. [50] | ***** | ***** | ***** | ***** |  | ***** | ***** | ***** | 7 |
| Junge & Dvorak, [28] | ***** | ***** | ***** | ***** | ***** | ***** | ***** | ***** | 6 |
| Junge & Dvorak, [41] | ***** | ***** | ***** | ***** |  | ***** | ***** |  | 6 |
| Le Gall et al. [34] | ***** |  |  | ***** | ***** | ***** | ***** | ***** | 5 |
| Lislevand et al. [43] | ***** | ***** |  | ***** |  | ***** | ***** | ***** | 7 |
| Mann et al. [48] | ***** |  | ***** | ***** | ***** | ***** | ***** | ***** | 8 |
| McGuine et al. [29] | ***** | ***** | ***** | ***** |  | ***** | ***** | ***** | 6 |
| Mendez-Rebolledo et al. [49] | ***** | ***** | ***** | ***** |  | ***** | ***** |  | 6 |
| Moller et al. [44] | ***** | ***** | ***** | ***** | ***** | ***** | ***** | ***** | 7 |
| Owoeye et al. [56] | ***** | ***** | ***** | ***** |  | ***** | ***** |  | 8 |
| Pluim et al. [31] | ***** | ***** | ***** | ***** |  | ***** | ***** |  | 6 |
| Schiff et al. [38] | ***** | ***** | ***** | ***** |  | ***** | ***** | ***** | 6 |
| Sokka et al. [32] | ***** | ***** | ***** | ***** | ***** | ***** | ***** | ***** | 7 |
| Soligard et al. [40] | ***** | ***** |  | ***** |  | ***** | ***** | ***** | 7 |
| Sprouse et al. [30] | ***** | ***** | ***** | ***** |  | ***** | ***** | ***** | 6 |
| Steffen et al. [39] | ***** | ***** |  | ***** | ***** | ***** | ***** | ***** | 8 |
| Watson et al. [53] | ***** | ***** | ***** | ***** |  | ***** | ***** | ***** | 6 |
| Wedderkopp et al. [45] | ***** | ***** | ***** | ***** |  | ***** | ***** | ***** | 6 |
| Westin et al. [55] | ***** | ***** | ***** | ***** |  | ***** | ***** | ***** | 7 |
|  |  |  |  |  |  |  |  | **Mean** | 7 |
|  |  |  |  |  |  |  |  | **SD** | 1 |
